# ISO 10993 biological evaluation of novel hemostatic powder – 4SEAL®

**DOI:** 10.1186/s40824-022-00258-6

**Published:** 2022-04-05

**Authors:** Lukasz Szymanski, Kamila Golaszewska, Anna Wiatrowska, Monika Dropik, Pawel Szymanski, Bartosz Gromadka, Patrycja Krakowiak, Justyna Wierzchowska, Damian Matak

**Affiliations:** 1 Department of Molecular Biology, Institute of Genetics and Animal Biotechnology, Polish Academy of Science, Postępu 36A, 05-552 Magdalenka, Poland; 2 European Biomedical Institute, Nalkowskiej 5, 05-410 Jozefow, Poland

**Keywords:** 4SEAL hemostatic powder, Biocompatibility, Biological evaluation, ISO 10993, Hemostatic, Adhesion prevention, Polysaccharide

## Abstract

**Background:**

Hemostasis plays a crucial role during every surgery allowing for a bloodless operating field. Fast and effective surgery leads to a reduced risk of postoperative complications. One of the latest methods for achieving homeostasis is using natural polysaccharide-based hemostatic powders. The study aimed to evaluate the biocompatibility according to the ISO 10993 standards of 4SEAL® Hemostatic powder.

**Methods:**

Chemical characterization (Headspace GC-MS, GC-MS, and ICP-MS), cytotoxicity, genotoxicity (MLA and AMES), endotoxin contamination, sensitization potential, intracutaneous reactivity, acute and subacute systemic toxicity with implantation, and pyrogenicity were evaluated to investigate the biocompatibility of the 4SEAL® Hemostatic powder. Studies were conducted according to ISO 10993 standards.

**Results:**

The biocompatibility requirements according to ISO 10993-1 for 4SEAL® Hemostatic powder were met. Based on the conducted in-vitro studies, 4SEAL® Hemostatic powder shows a non-cytotoxicity and non-mutagenic potential. Also, the results of in vivo studies of 4SEAL® Hemostatic powder shows no signs of toxicity, non-sensitizing, non-irritating, and no pyrogenicity potential. In the chemical characterization of 4Seal® Hemostatic Powder, no compounds were identified above Analytical Evaluation Threshold (AET) and no elements with concentrations higher than element-specific PDE [μg/day] were detected.

**Conclusions:**

4SEAL® Hemostatic powder is a promising new hemostatic agent with a wide range of potential applications and excellent biocompatibility.

**Supplementary Information:**

The online version contains supplementary material available at 10.1186/s40824-022-00258-6.

## Introduction

Hemostasis plays a crucial role throughout every surgery by allowing for a bloodless operating field. Fast and effective surgery leads to a reduced risk of postoperative complications. However, intraoperative bleeding or postoperative hemorrhage is a significant concern, and it presents significant perioperative morbidity [1]. In addition, hemostasis failure leads to prolonged hospitalization, increased infection risk, and potential further surgical interventions. Hemostasis is a process that prevents and stops bleeding. Biologically, it is a complex process requiring precisely coordinated activation of platelets and plasma clotting factors to form a platelet-fibrin clot [2]. It can be divided into two distinct processes, primary and secondary hemostasis. Primary hemostasis results in the formation of soft platelet plugs, which are stabilized and cross-linked during secondary hemostasis. Of central importance in both primary and secondary hemostasis is the activation of the clotting cascade, which can be broken down into two pathways [3]. The intrinsic pathway is activated by collagen, which is exposed when a blood vessel is damaged. The extrinsic pathway is similarly activated by tissue damage and the resultant release of tissue factors. Finally, the intrinsic and extrinsic pathways converge into the common pathway, which begins with the conversion of Factor X to Xa, and ultimately results in the conversion of prothrombin to thrombin, which is integral in clot stabilization via fibrin [4, 5]. An appropriate method for achieving hemostasis depends on various factors such as location, visibility, the possibility of access, and bleeding intensity. The conventional methods include electrocoagulation, suturing, and pressure application. On the other hand, natural polysaccharide-based hemostatic powders gained popularity due to their ease of application and effectiveness in controlling mild to moderate bleeding.

Biocompatibility, as described in ISO 10993-1, is an ability of a medical device or material to perform with an appropriate host response in a specific application. The term is used to describe the potential biological risks associated with the use of any medical device in a clinical situation. Finally, biocompatibility is the basis for the determination of the safety of a medical device [6]. The use of non-biocompatible medical devices may lead to significant adverse effects, including systemic toxicity and multiple organ failure. The ultimate goal of biocompatibility testing is to mitigate the risk of medical devices however, within the limits of relevant regulations, the biocompatibility results for the product can be on either end of the allowed spectrum. The biocompatibility results for any tested product correlate with the risk of clinical use of the medical device, therefore the better the biocompatibility results, the lower the risk of occurrence of adverse effects. The 4SEAL® Hemostatic powder is a class III medical device, which means it is supposed to remain in the patient’s body and be dissolved and utilized by the patient’s enzymatic apparatus. This means that the contact time between the device and the patient’s tissue is categorized as prolonged. Therefore, the concern of its biocompatibility is very significant. The biological response to such a medical device needs to be investigated with additional attentiveness to ensure the patient’s safety through the procedure. Thus, the present study investigates the biocompatibility of 4SEAL® Hemostatic powder according to the ISO 10993.

### Study product

**Fig. 1:**
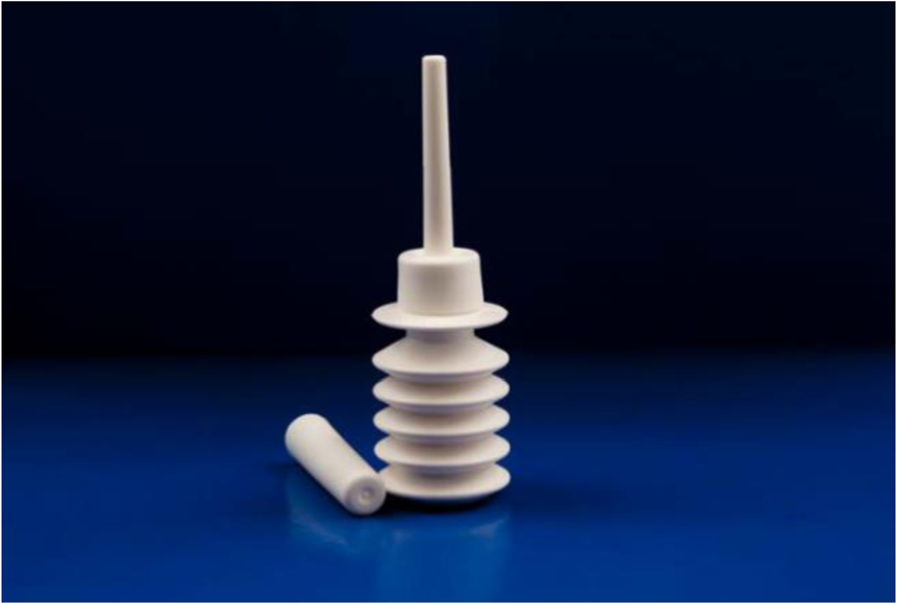
Photo of 4SEAL® Hemostatic powder

4SEAL® Hemostatic powder (Grena Biomed LTD) is a hemostatic medical device consisting of natural polysaccharide-based fine powder particles and a delivery applicator. The product is ready to use, thus can be used immediately. 4SEAL® Hemostatic powder is used to stop bleeding during surgical procedures or traumatic injuries. The ultra-hydrophilic particles are derived from purified starch. 4SEAL® Hemostatic powder absorbs water rapidly from the blood, which causes a high concentration of thrombocytes, leukocytes, red blood cells, and coagulation proteins, thus inducing instantly primary hemostasis and accelerating the blood clotting process. For adhesion prevention, 4SEAL® Hemostatic powder is indicated when the formation of postoperative adhesion is to be prevented after surgical interventions in cavities covered by mesothelium. The product is plant-based and contains no animal or human-derived materials. The absorption process of 4SEAL® Hemostatic powder begins immediately and is dependent on several factors, including the amount applied and the site of use. Generally, its complete absorption takes a few days. The studied product is shown in Fig. 1.

## Material and methods

Detailed data for every experiment and additional information regarding materials and methods are presented in Supplement 1. Cell lines were purchased from ATCC, reagents for cell culture were purchased from Thermo Fisher Scientific, Poland, and all chemical compounds were purchased from Sigma, Poland, unless otherwise indicated.

### Statistical analysis

All results were presented as the mean ± standard deviation (SD). Statistical evaluation of MTT cytotoxicity and sensinization studies was performed using the one-way ANOVA with Bonferroni’s multiple comparisons test. Subacute toxicity combined with implantation results were analyzed using two-tailed heteroscedastic T-test. GraphPad Prism software (version 9.3.1; GraphPad Software, Inc., La Jolla, CA, USA) was used for all evaluations. *p* <  0.05 was considered statistically significant.

### Extraction

Extractions were performed according to ISO 10993-12 [7]. Extraction media for each experiment were chosen based on the appropriate ISO norm for the study. Briefly, the extractions were prepared by incubating the test material with a suitable extraction medium at 50 ± 1 °C for 72 ± 2 h for AMES, ICP-MS, GC-MS, and LC-MS studies or at 37 ± 1 °C for 72 ± 2 h for other studies unless otherwise indicated. The extraction volume was derived from Table 1 – Standard surface areas and extract liquid volumes, ISO 10993-12 and determined at 0.2 g/mL [7]. The extracts were not centrifuged, filtered, or otherwise altered prior to dosing. The extract was clear without the presence of any particulates. The extracts were used within 24 h of preparation. Since the material absorbs vehicles, the extraction vehicle that each 0.1 g of material absorbs was determined. The additional volume of the extraction vehicle was added to the mixture during the extraction.

**Table 1:** Positive controls list for AMES test

Strain	Substance	
	Without S9 fraction	With S9 fraction
*Salmonella typhimurium* TA98	2-nitrofluorene (2-NF)	2-aminoanthracene (2-AA)
*Salmonella typhimurium* TA100	4-nitroquinoline N-oxide (4-NQO)	2-AA
*Salmonella typhimurium* TA1535	N4-aminocytidine (N4-ACT)	2-AA
*Salmonella typhimurium* TA1537	9-aminoacridine (9-AA)	2-AA
*E.coli* WP2 uvrA [pKM101]	4-NQO	2-aminofluorene (2-AF)

### Chemical characterization

Per ISO 10993-18, a semi-quantitative analysis of VOC (volatile organic compound) in product and SVOC (semi-volatile organic compound) in 4SEAL® Hemostatic powder water extract was performed [8]. QP2010 Ultra gas chromatograph and QP-5000 mass spectrometer (Shimadzu) were used for analysis. In addition, quantitative analysis of elements concentration in 4SEAL® Hemostatic powder water extract was performed using NexION 300D (Perkin Elmer). As per ICH Q3D (R1), the concentration of the following elements was examined: Cd, Pb, As, Hg, Co, V, Ni, Tl, Au, Pd, Ir, Os, Rh, Ru, Se, Ag, Pt, Li, Sb, Ba, Mo, Cu, Sn, Cr [9].

For the analysis of VOCs and SVOCs, the Analytical Evaluation Threshold (AET) [8] was calculated according to the following formula:

$$ AET\ \left(\frac{\mu g}{ml}\right)=\frac{DBT\ast \frac{A}{BC}}{UF} $$

where:

A – number of devices extracted.

B – volume of the extract.

C – clinical exposure to medical device per day.

DBT – the dose based threshold (TTC).

UF – uncertainty factor (according to ISO 10993-18, a value of 2 is assumed for semiquantitative tests and a value of 1 for quantitative tests) [8].

Assuming an uncertainty factor of 2, ten 5 g bottles of 4SEAL® Hemostatic powder per patient usage, and Threshold of Toxicological Concert of 120 μg/day, the AET was calculated to be 0.240 μg/mL.

For the analysis of each element concentration, Total Element Exposure (TEE) [8] was calculated assuming device patient usage of 50 g per day. TEE was calculated as follows:

$$ TEE\left[\mu g\right]=\frac{Element\ concentration\ast number\ of\ devices\  per\  patient}{numberofdevicesused\ for\ extraction\ast \frac{1000}{extraction\ volume}} $$

### MTT cytotoxicity

Cytotoxicity was evaluated quantitatively using the MTT method based on the ISO 10993-5 and ISO 10993-12 [7, 10]. Briefly, 4SEAL® Hemostatic powder was extracted in single strength MEM at 37 ± 1 °C for 24 ± 1 h. Following the extraction, quadruplicate monolayers of L-929 mouse fibroblast cells were dosed with: 100, 50, 33, and 25% extracts and incubated at 37 ± 1 °C, 5 ± 1% CO_2_, 95% humidity for 24 ± 1 h. Following the incubation, 50 μL of the MTT solution, prepared just before use, were dispended in each well and incubated for 120 min at 37 ± 1 °C, 5 ± 1% CO_2_, 95% humidity. Following the incubation, MTT solution was replaced with 100 μL isopropanol and incubated for 10 min in 37 ± 1 °C, 5 ± 1% CO_2_, 95% humidity. Finally, the optical density was measured by absorption at 570 nm (650 nm reference). The percent viability was determined from the blanks.

### Genotoxicity

Extraction of 4SEAL® Hemostatic powder for genotoxicity studies.

The amount of extractables was assessed by a pre-experiment “Determination of Extractables” according to ISO 10993-3 [11]. Based on the results, Method C – extraction according to ISO 10993-12 was chosen [7]. The extraction was conducted using an appropriate extraction vehicle.

### Mouse lymphoma assay - MLA

Based on the ISO 10993-3, ISO 10993-12, ISO 10993-33, and OECD Test No 490, the 4SEAL® Hemostatic powder genotoxicity was evaluated using Mouse Lymphoma Assay [7, 11–13]. In short, mycoplasma-free L5178Y TK+/− 3.7.2C cells were cultured (37 ± 1 °C, 5 ± 1% CO_2_, 95% humidity) in the F10 medium to the sufficient number and cleansed using THMG for 1 day and then THG for 2 days (complete formulation of THMG and THG is presented in Supplement Data). The cleansed cells were then used in the experiment. 6 * 10^5^ (for 4 h treatment) or 4 * 10^5^ (for 24 h treatment) cells were exposed to the 10 ml of the 100% sample extract (worst case scenario), appropriate positive control, or negative control. The cells treatment was performed for 4 h with and without the presence of 1% liver Aroclor-induced S9 fraction and for 24 h without S9 fraction in 37 ± 1 °C, 5 ± 1% CO_2_, 95% humidity. A duplicate test sample, duplicate negative control, and one positive control was prepared for each condition. After the 4 h treatment, the cells were centrifuged and washed twice with fresh medium and then resuspended in the 20 ml of the F10 medium. After additional 20 h, the cells were counted and resuspended in a fresh F10 medium at the concertation of 2 * 10^5^. The cells were incubated for 24 h (37 ± 1 °C, 5 ± 1% CO_2_, 95% humidity) and recounted. After the 24 h treatment, the cells were counted, washed twice, and resuspended in F10 fresh medium at the concertation of 2 * 10^5^. The cells were incubated for 24 h (37 ± 1 °C, 5 ± 1% CO_2_, 95% humidity) and recounted. The number of cells counted was used to calculate total suspension growth (TSG) according to ISO 10993-33. After the expression period, the cell’s relative plating efficiency (RPE; percentage plating efficiency of the test group in relation to the negative control) was determined by seeding a statistical number of 1.6 cells/well in two 96-well plates in F20 medium (corresponding to a density of 8 cells/ml as per OECD Test No 490). The cells were incubated for 14 days at 37 ± 1 °C in the humidified atmosphere in the presence of 5% CO_2_. Analysis of results was based on the number of cultures without cell growth compared to the total number of cultures seeded. Relative suspension growth and relative total growth (RSG and RTG; RTG = RSG x RPE / 100) of the treated cell cultures were calculated according to the ISO 10993-33. RTG measures relative (to the vehicle control) growth of test cultures during the treatment, two-day expression, and mutant selection cloning phases of the test. The RSG of each test culture is multiplied by the relative cloning efficiency of the test culture at the time of mutant selection and expressed relative to the cloning efficiency of the negative/solvent control [13]. RPE is the percentage of inoculated cells that give rise to colonies relative to the control where the absolute plating efficiency is arbitrarily set as 100 [14].

Additionally, cultures were seeded in a selective medium. Cells from each experimental group were seeded in four 96-well plates at a density of 2000 cells/well in 200 μl selective F20 medium with TFT. The plates were scored after an incubation period of 14 days at 37 ± 1 °C in the humidified atmosphere in the presence of 5% CO_2_. Small colonies were counted separately. Small colonies are defined as less than a quarter of the diameter of the well, while large colonies are more than a quarter of the diameter of the well. The mutation frequencies were calculated from the data obtained from cultures used for the plaiting efficiency (cultures with non-selective medium) and those used for selection (cultures with selective medium) according to the following formula:

$$ Mutant\ frequency\  per\ {10}^6 cells=\frac{\frac{-\ln \left( empty\ wells\ of\ mutant\ selection\ plates/384\right)}{2000}}{\frac{-\ln \left( empty\ wells\ of\ non\ selection\ plates/192\right)}{1.6}}\ast {10}^6 $$

### Bacterial reverse mutation test - AMES

Genotoxicity of 4SEAL® Hemostatic powder was evaluated using commercially available Bacterial Reverse Mutation Test AMES Penta 2 (Xenometrix) according to ISO 10993-3, ISO 10993-12, ISO 10993-33, and OECD Test No. 471 [7, 11, 12, 15].

Bacteria were exposed to the 25 μl of full strength extracts of the test material as well as positive (Table 1) and negative controls for 135 min in a medium containing sufficient histidine (*S. typhimurium*) or tryptophan (*E. coli*) to support approximately two cell divisions. The volume of extract added was based on the ISO 10993-33 and kit manufacturers’ documentation. After exposure, the cultures were diluted in a pH indicator medium lacking histidine or tryptophan and aliquoted into 48 wells of a 384-well plate. After two days, cells that have undergone reversion to amino acid prototrophy grow into colonies. Bacterial metabolism reduces the pH of the medium, changing the color of that well. The number of wells containing revertant colonies were counted for each group (test article and positive control) and compared to a solvent (negative) control. Samples were prepared in triplicate to allow for statistical analysis of the data. The mutagenic potential of samples was assessed directly and in the presence of 4.5% of liver Aroclor-induced S9 fraction. Baseline, fold increase over baseline value, and binomial B-value were calculated using an excel spreadsheet provided by the manufacturer. The baseline is calculated as the negative control’s mean plus standard deviation. Fold increase over baseline is calculated by dividing the mean number of positive wells for a sample by the baseline value. The binomial B-value indicates the probability that spontaneous mutation events occur. For example, a binomial B-value ≥0.99 indicates that chances are ≤1% that this Result is due to spontaneous mutation. If both fold increase ≥2 and binomial B-value ≥0.99 occur for a test sample in specific conditions (strain, +/− S9 fraction), it should be considered mutagenic.

### Endotoxins

**Fig. 2:**
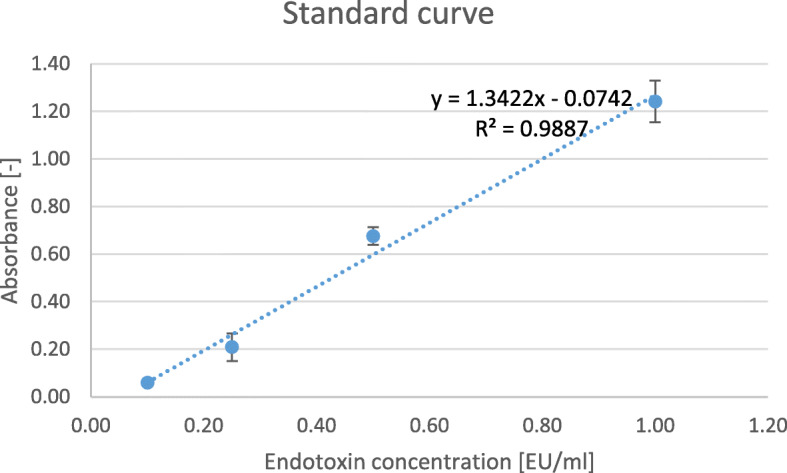
Endotoxins concentration standard curve using using Pierce Chromogenic Endotoxin Quant K, prepared according to the manufactuter’s instruction. R^2^ = 0.9887

Endotoxins were measured using Pierce Chromogenic Endotoxin Quant K, which is in regard to 85. Bacterial Endotoxin Test, U.S. Pharmacopoeia [16]. The 4SEAL® Hemostatic powder was extracted in water for injection using an extraction ratio of 0.2 g/mL. According to the manufacturer’s instruction, the standard curve was prepared (R^2^ = 0.9887) and is shown in Fig. 2. Internal validation of the experiment was performed by spiking the samples with 0.05 EU/mL of endotoxin. The unspiked and spiked samples were assayed to determine the respective endotoxin concentrations. For the test to be valid, the difference between the two calculated endotoxin values should equal the known (0.5 EU/mL) concentration of the spike ±25%.

### Sensitization

The sensitization potential of the 4SEAL® Hemostatic powder was analyzed according to the ISO 10993-10 using the Local Lymph Node Assay (LLNA) [17]. Briefly, 4SEAL® Hemostatic powder was extracted using Acetone: Olive Oil 4:1. 15 adult, albino, healthy house mice (*Mus musculus*) of BALB/c strain were randomly assigned to solvent control, positive control, or study group. The test samples, solvents control, and positive control were applied to the dorsal side of both ears of designated mice at a dose of 25 μl / day for three consecutive days. 48 h after the last extract application, mice were injected with 0.5 mL of BrdU (10 mg/mL) in phosphate-buffered saline (PBS) solution intra-peritoneally. 24 h after BrdU injection, animals were sacrificed, and auricular lymph nodes were harvested. A single-cell suspension of lymph node cells (LNC) was prepared from each mouse by gentle mechanical disaggregation through a disposable μ70 nylon cell strainer. In each case, the target volume of the LNC suspension was adjusted to 15 mL. The incorporation of BrdU was measured using the Colorimetric BrdU Cell Proliferation ELISA Kit (Abcam) according to the manufacturer’s recommendations. 50 μL of each LNC was transferred in triplicate into a 96-well cell culture plate. PBS was used as a blank control. The colored reaction product was quantified using the μQuant spectrophotometer with dual-wavelength of 450/550 nm. BrdU labeling index was calculated according to the formula:

$$ \mathrm{BrdU}\ \mathrm{labelling}\ \mathrm{index}=\left({\mathrm{ABS}}_{\mathrm{em}}-\mathrm{ABS}\ {\mathrm{blank}}_{\mathrm{em}}\right)-\left({\mathrm{ABS}}_{\mathrm{ref}}-\mathrm{ABS}\ {\mathrm{blank}}_{\mathrm{ref}}\right). $$

em - emission wavelength; ref. - reference wavelength.

For the test sample and positive control Sensitization Index (SI) was calculated according to the formula:

$$ SI=\frac{BrdU\ labelling\ index\ of\ the\ sample}{BrdU\ labelling\ index\ of\ the\ negative\ control} $$

For the test to be valid, the SI of positive control (PC) must be higher than 2.

### Intracutaneous reactivity

The study was conducted according to ISO 10993-10 [17]. The test article was extracted using Sodium Chloride and Cottonseed Oil as described in the Extraction section of material and methods. According to ISO 10993-18:2020, an exaggerated extraction was performed by immersing the test item in the extraction medium [8]. According to the ISO 10993-12:2021, the extraction volume was set to reach a mass/volume ratio of 0.2 g/mL. Samples were incubated at 37 ± 1 °C for 72 ± 2 h [7]. Before the treatment, the fur on the animal’s back on both sides of the spinal column was closely clipped over a sufficiently large test area, avoiding mechanical irritation and trauma. Then, 0.2 ml of the polar (Sodium Chloride) and non-polar (Cottonseed Oil) extracts were injected intracutaneously at five sites on one side of each rabbit. Similarly, 0.2 ml of the polar and non-polar solvent controls were injected intracutaneously on five sites of the contralateral side of each rabbit. The animals were observed immediately after injection, 24 ± 2, 48 ± 2, and 72 ± 2 h after the treatment to evaluate the signs of local reaction. Injection sites were examined for evidence of any tissue reaction such as erythema, oedema, and eschar. Tested and control sites were scored according to Table 2 below.

**Table 2:** The grading system for intracutaneous (intradermal) reactions

Reaction	Numerical grading
**Erythema and eschar formation**	
No erythema	0
Very slight erythema (barely perceptible)	1
Well defined erythema	2
Moderate erythema	3
Severe erythema (beet redness) to eschar formation preventing grading of erythema	4
**Oedema formation**	
No oedema	0
Very slight oedema (barely perceptible)	1
Well defined oedema (edges of area well defined by define raising)	2
Moderate oedema (edges raised approximately 1 mm)	3
Severe oedema (raised more than 1 mm and extended beyond exposure area)	4

After the 72 ± 2 h grading, all erythema grades plus oedema grades (at 24 ± 2 h, 48 ± 2 h, and 72 ± 2 h) are separately summed for each test sample or blank for each animal. To calculate the score of a test sample or blank on each animal, divide each total by 15 (3 scoring time points × 5 test or blank sample injection sites). To determine the overall mean score for each test sample and each corresponding blank, add the scores for the three animals and divide them by three. The final test sample score is obtained by subtracting the blank score from the test sample score. The acceptance criteria are met if the final test score is 1.0 or less.

### Acute systemic toxicity

The study was conducted according to ISO 10993-10 [17]. Four groups of 5 animals were injected with 50 ml/kg of Sodium Chloride extract, Cottonseed Oil extract, the polar and non-polar solvent controls. Polar and non-polar extracts and solvent controls were injected intraperitoneal. Animals underwent a clinical examination and were weighted 24 ± 2 h, 48 ± 2 h, 72 ± 2 h after injection. 72 ± 2 h after injection, animals were euthanized.

### Subacute toxicity combined with implantation

Based on ISO 10993-6 and ISO 10993-11, 4SEAL® Hemostatic powder was evaluated for subacute toxicity combined with implantation using Starsil® Hemostat as reference material [18, 19]. Cannulas from peripheral venous access catheters were divided into 10 mm pieces. Pieces were divided into two groups – the control and test group. Each piece from the control group was filled with approx. 0.3 g of Starsil® Hemostat while each piece from the test group was filled with 0.3 g of 4SEAL® Hemostatic powder. Before animal treatment, the fur on the animal’s back was closely clipped over a sufficiently large test area, avoiding mechanical irritation and trauma. Place of implantation was disinfected by iodine solution. Surgery was performed under general anesthesia using isoflurane when animals received analgesic – subcutaneously injected butorphanol (2 mg/kg). An incision was made on the skin in a paraspinal line. Implants were placed on both flanks of the animal at equal intervals, in separate pockets in subcutaneous tissue. Eight pieces per rat of test or control article were implanted. Control and test materials had contact with surrounding tissue only in the base of cylindrical implants. Wounds were closed using non-resorbable threads. After implantation, each animal was injected subcutaneous meloxicam (1 mg/kg). Animals were housed separately for a week until wounds were healed. For 3 days after implantation, each animal was injected subcutaneous meloxicam (1 mg/kg). Animal’s weight was observed 1, 2, 3, 7, 14, 21, 28 days after implantation. On the 27th day of the experiment, urine samples were collected. After 28 days, animals were premedicated with Ketamine/Xylazine (100 mg/kg – Ketamine, 10 mg/kg – Xylazine) to collect blood samples and then killed by CO2. At the end of the exposure period, routine hematology and clinical chemistry were conducted on all animals. Animals were anesthetized with Ketamine/Xylazine, and blood was drawn into tubes with K2-EDTA for hematology and heparin for clinical chemistry. Hemoglobin, PCV, RBC, reticulocytes, thrombocytes, and total WBC were determined with a hematology analyzer (Scil, Germany). Plasma concentrations of glucose, ALP, ALAT, ASAT, GGT, total protein, albumin, urea, creatinine, total bilirubin, total cholesterol, triglycerides, phospholipids, Ca2+, Na+, K+, Cl - and inorganic phosphate were determined using a biochemical analyzer (Fujifilm, Poland).

According to 4SEAL® Hemostatic powder Instruction For Use, maximal patient exposition to the hemostatic powder is 50 g. Statistical human weight is 60 kg. Each animal was implanted with 8 implants containing 0.3 g of hemostatic powder. The dose of test articles for a single rat was more than 10x of the maximal human dose.

#### Gross necropsy

After animal euthanasia, a gross necropsy was performed on all animals. The following organs were weighed (paired organs together) after dissection: adrenals, brain, lungs, heart, kidneys, liver, ovaries, spleen, testes. The organ-to-body weight ratios (relative organ weights) were calculated from the rats’ absolute organ weights and the terminal body weight.

Samples of the weighed organs and the colon, lymph nodes, skin, lungs, mammary gland, peripheral nerve (sciatic), esophagus, parathyroid, pituitary, prostate, rectum, small intestines (duodenum, ileum, jejunum), sternum with bone marrow, stomach, thyroid, trachea with bronchi, urinary bladder, uterus, vagina, places of implantation and all gross lesions were preserved in a neutral aqueous phosphate-buffered 4% solution of formaldehyde. Histopathologic analysis from organs: brain, lungs, heart, liver, kidneys, adrenals, ovaries/testis, sternum, muscle, the skin was conducted on 5 μm sections of paraffin-embedded tissues, stained with hematoxylin and eosin, of the preserved organs from two representative animals per sex from control and test group by light microscopy. Each place of subcutaneous implantation was examined under a microscope and evaluated based on the guidelines provided in Table 3.

**Table 3:** Guidelines of histological evaluation system of place of implantation – tissue response

Histologic feature	Score				
	0	1	2	3	4
Inflammatory cell type/response — Polymorphonuclear cells — Lymphocytes	0	Rare, 1 to 5/hpf ^a^	Rare,5 to 10/hpf ^a^	Moderate infiltrate	Marked infiltrate
Plasma cells					
Macrophages/gitter cells					
Multinucleated giant cells MGC)	0	Rare, 1 to 2/hpf	Rare, 3 to 5/hpf		
Necrosis	0	Minimal	Mild	Moderate infiltrate	Marked
Neovascularization	0	Minimal capillary proliferation, focal, 1 to 3 buds	Groups of 4 to 7 capillaries with supporting fibroblastic structures	Broad band of capillaries with supporting fibroblastic structures	Extensive band of capillaries with supporting fibroblastic structures
Fibrosis	0	Narrow band	Moderately thick band	Thick band	Extensive band
Astrocytosis/fatty infiltration					

^a^ hpf = high-powered (400x) field

### Pyrogenicity

#### Rabbit selection

Rabbits used for the study were submitted to a negative pyrogen test within 14 days preceding the test (with a rest period of a minimum of 3 days following the negative pyrogen test).

#### Determination of the initial temperature

Previously weighted rabbits were placed in a restrainer, and a thermometric rectal probe was inserted at not less than 7.5 cm but not more than 9 cm. The temperature of each rabbit was recorded every 30 min for 90 min before injection. The rabbits which showed a temperature variation two successive readings higher than 0.2 °C during the initial temperature determination or which showed a temperature higher than 39.6 °C or lower than 38.2 °C were not injected. The initial temperature of each rabbit was determined as the mean of two temperatures recorded at intervals of 30 min before the injection. In the group, the difference between the tree initial temperatures did not exceed 1 °C.

#### Rabbit injection and follow up

After extraction, the tested solution was warmed to about 38.5 °C and injected intravenously via the marginal ear vein at a dose of 10 ml/kg of body weight. The temperature of each rabbit was recorded every 30 min for 3 h after injection. The maximum rise (compared to the initial temperature) of each rabbit was determined at the end of the test. Acceptance criteria for the test are presented in Table 4.

**Table 4:** Criteria of acceptance for the pyrogenicity test

Number of rabbits	Product passes if the summary response does not exceed	The product fails if the summary response exceeds
3	1.15 °C	2.65 °C
6	2.80 °C	4.30 °C
9	4.45 °C	5.95 °C
12	6.60 °C	6.60 °C

## Results

### Chemical characterization

Determination of extraction conditions for exhaustive extraction of 4SEAL® Hemostatic powder has revealed that isopropanol and hexane cause product degradation and vehicle color change. Therefore, only water extract was analyzed as per ISO 10993-18.

No VOCs above AET were identified.

No SVOCs above AET were identified.

No elements above the limit were identified. The results are presented in Table 5.

**Table 5:** ICP MS results of 4SEAL® Hemostatic powder

Analyzed element	Limit of detection (LOD) [μg/L]	Result [μg/L]	Total Element Exposure [μg]	Parenteral PDE [μg/day]
**Cd**	1	< 1	< 0.25	2
**Pb**	2.5	< 2.5	< 0.625	5
**As**	30	< 30	< 7.5	15
**Hg**	1	< 1	< 0.25	3
**Co**	2.5	< 2.5	< 0.625	5
**V**	24	< 24	< 6	10
**Ni**	10	< 10	< 2.5	20
**Tl**	5	< 5	< 1.25	8
**Au**	5	< 5	< 1.25	100
**Pd**	5	< 5	< 1.25	10
**Ir**	5	< 5	< 1.25	10
**Os**	5	< 5	< 1.25	10
**Rh**	5	< 5	< 1.25	10
**Ru**	5	< 5	< 1.25	10
**Se**	10	< 10	< 2.5	80
**Ag**	5	< 5	< 1.25	10
**Pt**	5	< 5	< 1.25	10
**Li**	10	< 10	< 2.5	250
**Sb**	10	< 10	< 2.5	90
**Ba**	10	< 10	< 2.5	700
**Mo**	10	< 10	< 2.5	1500
**Cu**	130	< 130	< 32.5	300
**Sn**	10	< 10	< 2.5	600
**Cr**	60	< 60	< 15	1100

### MTT cytotoxicity

**Fig. 3:**
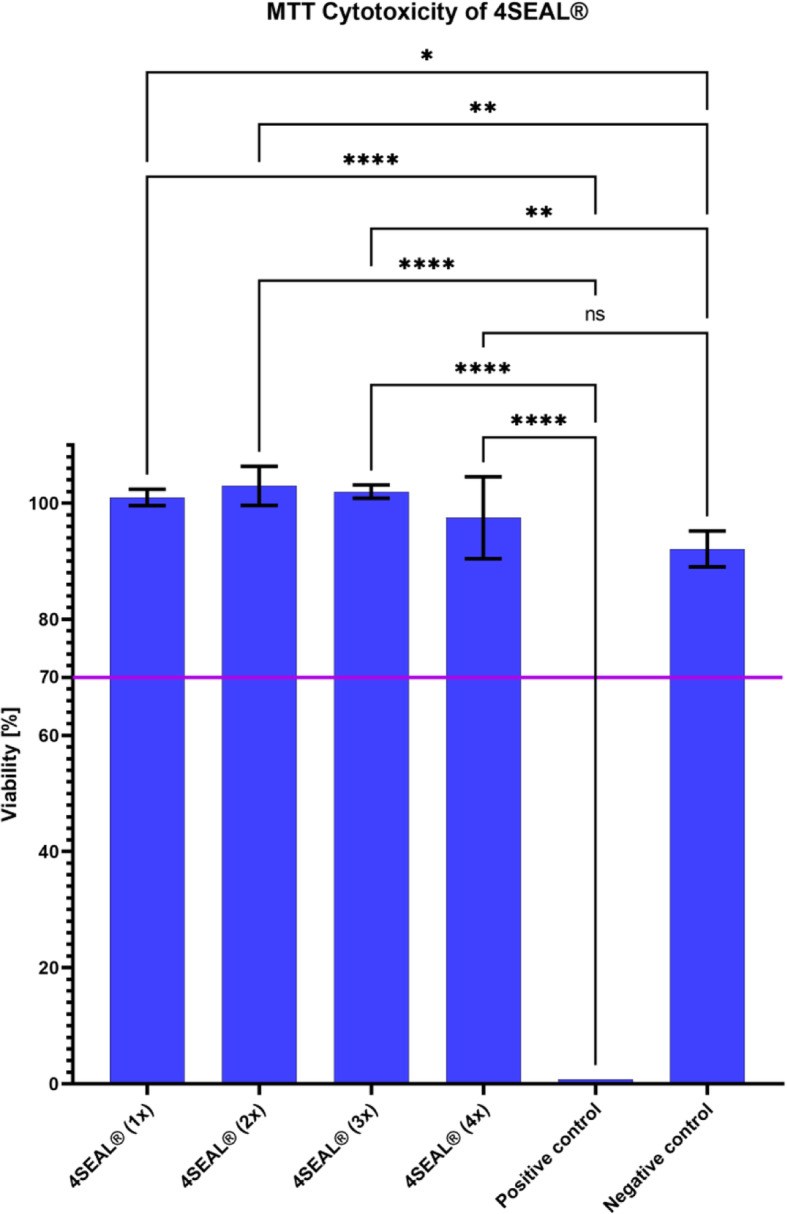
MTT Cytotoxicity of 4SEAL® Hemostatic Powder. No cytotoxic potential of cell culture medium extract and its dilutions to L-929 mouse fibroblasts was observed. Horizontal line at 70% represents a cut off below which the extract is considered cytotoxic as per ISO 10993-5

4SEAL® Hemostatic powder cell culture medium extract and its dilutions showed no cytotoxic potential to L-929 mouse fibroblasts using the quantitative MTT method. The cellular response observed from the positive and negative controls, systemic cell seeding errors, and absolute value of optical density confirmed the suitability of the test system. Results of cytotoxicity testing are presented in Table 6 and Fig. 3.

**Table 6:** Cytotoxicity results

Material	Percent Viability [%]	System Suitability
Positive control	0.77	Met criteria
Negative control	92.10	Met criteria
4SEAL® (1x)	101.05	No Cytotoxic Potential
4SEAL® (2x)	102.79	No Cytotoxic Potential
4SEAL® (3x)	101.91	No Cytotoxic Potential
4SEAL® (4x)	97.41	No Cytotoxic Potential
**Quality check of assay**	**Result**	**System Suitability**
Absolute value of optical density (OD_570_)	1.162	Met criteria
Systematic cell seeding errors	3.36%	Met criteria

### Genotoxicity (AMES and MLA)

#### Ames

Every strain employed in the test, both with and without S9 fraction, passed internal quality controls. 4SEAL® Hemostatic powder showed an unclear mutagenic effect only when exposed to the TA1535 strain with the presence of the S9 fraction. Results of cytotoxicity testing are presented in Table 7.

**Table 7:** AMES assay results

Strain	without S9			with S9		
	Baseline	Fold increase over baseline	Binomial B-value	Baseline	Fold increase over baseline	Binomial B-value
TA98	1.00	1.33	0.82	3.75	0.18	0.12
TA100	9.00	0.81	0.17	14.61	0.64	0.19
TA1535	3.91	0.68	0.32	1.00	17.00	1.00
TA1537	1.82	0.55	0.86	2.00	0.50	0.65
E.coli uvrA [pKM101]	9.39	0.46	0.08	10.0	0.40	0.00

Data has been analyzed and summarized in the Table 8 below. No precipitation or toxicity was observed in this study.

**Table 8:** AMES overall results

Strain	Mutagenic data points		Overall Result for sample	Solvent control		Positive control	
	w/o S9	with S9		w/o S9	with S9	w/o S9	with S9
**TA98**	No	No	Probably not mutagenic	**PASS**	**PASS**	**PASS**	**PASS**
**TA100**	No	No	Probably not mutagenic	**PASS**	**PASS**	**PASS**	**PASS**
**TA1535**	No	Yes	Unclear, needs further evaluation	**PASS**	**PASS**	**PASS**	**PASS**
**TA1537**	No	No	Probably not mutagenic	**PASS**	**PASS**	**PASS**	**PASS**
***E. coli*** **uvrA [pKM101]**	No	No	Probably not mutagenic	**PASS**	**PASS**	**PASS**	**PASS**

#### Mouse lymphoma assay (MLA)

A sample is considered mutagenic if the increase in MF is above the Global Evaluation Factor that equals 126 (*10^−6^) over the negative control. The acceptance criteria for MLA and summarized results of the reliability check of the assay are presented in Supplementary Materials. No precipitation or toxicity was observed in this study. MLA results are presented in Tables 9, 10, 11, 12, 13 and 14.

**Table 9:** Toxicity data, 4 h exposure, without metabolic activation

Sample	Number of cells seeded (*10^5)	Number of cells 24 h after treatment (*10^5)	Number of cells 48 h after treatment (*10^5)	Total suspension growth	Relative Suspension Growth (RSG) [%]	Plating Efficiency [%]	Relative Plating Efficiency (RPE) [%]	Relative Total Growth (RTG) [%]
NC1	3	11.02	9.03	16.59	100.00	93.59	100.00	100.00
NC2	3	10.56	9.11	16.03				
PC	3	8.62	7.67	11.02	67.56	65.80	70.31	47.50
4SEAL® 1	3	10.38	8.97	15.52	95.15	92.08	98.39	93.62
4SEAL® 2	3	10.62	9.02	15.97	97.89	87.96	93.99	92.01

*PC* positive control, *NC* negative control

**Table 10:** Toxicity data, 4 h exposure, with metabolic activation

Sample	Number of cells seeded (*10^5)	Number of cells 24 h after treatment (*10^5)	Number of cells 48 h after treatment (*10^5)	Total suspension growth	Relative Suspension Growth (RSG) [%]	Plating Efficiency [%]	Relative Plating Efficiency (RPE) [%]	Relative Total Growth (RTG) [%]
NC1	3	10.23	9.12	15.55	100.00	103.91	100.00	100.00
NC2	3	9.89	9.21	15.18				
PC	3	8.67	7.27	10.51	68.37	61.30	59.00	40.33
4SEAL® 1	3	9.78	8.93	14.56	94.73	86.64	92.58	87.70
4SEAL® 2	3	9.83	9.01	14.76	96.07	84.09	89.86	86.32

*PC* positive control, *NC* negative control

**Table 11:** Toxicity data, 24 h exposure, without metabolic activation

Sample	Number of cells seeded (*10^5)	Number of cells 24 h after treatment (*10^5)	Number of cells 48 h after treatment (*10^5)	Total suspension growth	Relative Suspension Growth (RSG) [%]	Plating Efficiency [%]	Relative Plating Efficiency (RPE) [%]	Relative Total Growth (RTG) [%]
NC1	2	11.18	9.73	130.95	100.00	90.82	100.00	100.00
NC2	2	10.95	9.62	129.57				
PC	2	8.58	8.72	72.67	55.79	64.87	71.43	39.85
4SEAL® 1	2	10.79	8.96	108.52	83.31	86.64	95.41	79.49
4SEAL® 2	2	10.61	9.11	106.93	82.09	87.96	96.85	79.51

*PC* positive control, *NC* negative control

**Table 12:** Mutagenicity data, 4 h exposure, without metabolic activation

Sample	Number of large colonies	Number of small colonies	Mutant frequency (*10^-6)	Small colonies [%]	Small colonies mutant frequency (*10^-6)	Mutagenicity
NC1	78	2	128.82	2.50	3.22	N/A
NC2	64	2	97.72	3.03	2.96	N/A
PC	130	60	518.83	31.58	163.84	Mutagenic
4SEAL® 1	64	2	102.40	3.03	3.10	Not Mutagenic
4SEAL® 2	58	3	98.34	4.92	4.84	Not Mutagenic

*PC* positive control, *NC* negative control

**Table 13:** Mutagenicity data, 4 h exposure, with metabolic activation

Sample	Number of large colonies	Number of small colonies	Mutant frequency (*10^-6)	Small colonies [%]	Small colonies mutant frequency (*10^-6)	Mutagenicity
NC1	85	2	128.94	2.30	2.96	N/A
NC2	80	2	111.01	2.44	2.71	N/A
PC	54	88	376.58	61.97	233.38	Mutagenic
4SEAL® 1	77	4	136.72	4.94	6.75	Not Mutagenic
4SEAL® 2	62	6	115.89	8.82	10.23	Not Mutagenic

*PC* positive control, *NC* negative control

**Table 14:** Mutagenicity data, 24 h exposure, without metabolic activation

Sample	Number of large colonies	Number of small colonies	Mutant frequency (*10^-6)	Small colonies [%]	Small colonies mutant frequency (*10^-6)	Mutagenicity
NC1	78	2	122.97	2.50	3.07	N/A
NC2	64	2	108.83	3.03	3.30	N/A
PC	130	90	655.71	40.91	268.25	Mutagenic
4SEAL® 1	64	2	108.83	3.03	3.30	Not Mutagenic
4SEAL® 2	58	3	98.34	4.92	4.84	Not Mutagenic

*PC* positive control, *NC* negative control

#### Endotoxin concentration

Endotoxin testing results per mL are presented in Table 15 and per 1 g finished device are presented in Table 16.

**Table 15:** Endotoxins concentration results

	4SEAL®	Spike
**Concentration [EU/mL]**	0.103	0.695

**Table 16:** Endotoxins concentration per device

	4SEAL®
**Endotoxin content per device (1 g) [EU]**	0.514

#### Sensitization

Each day animals were observed for signs of toxicity or skin irritation.

**Fig. 4:**
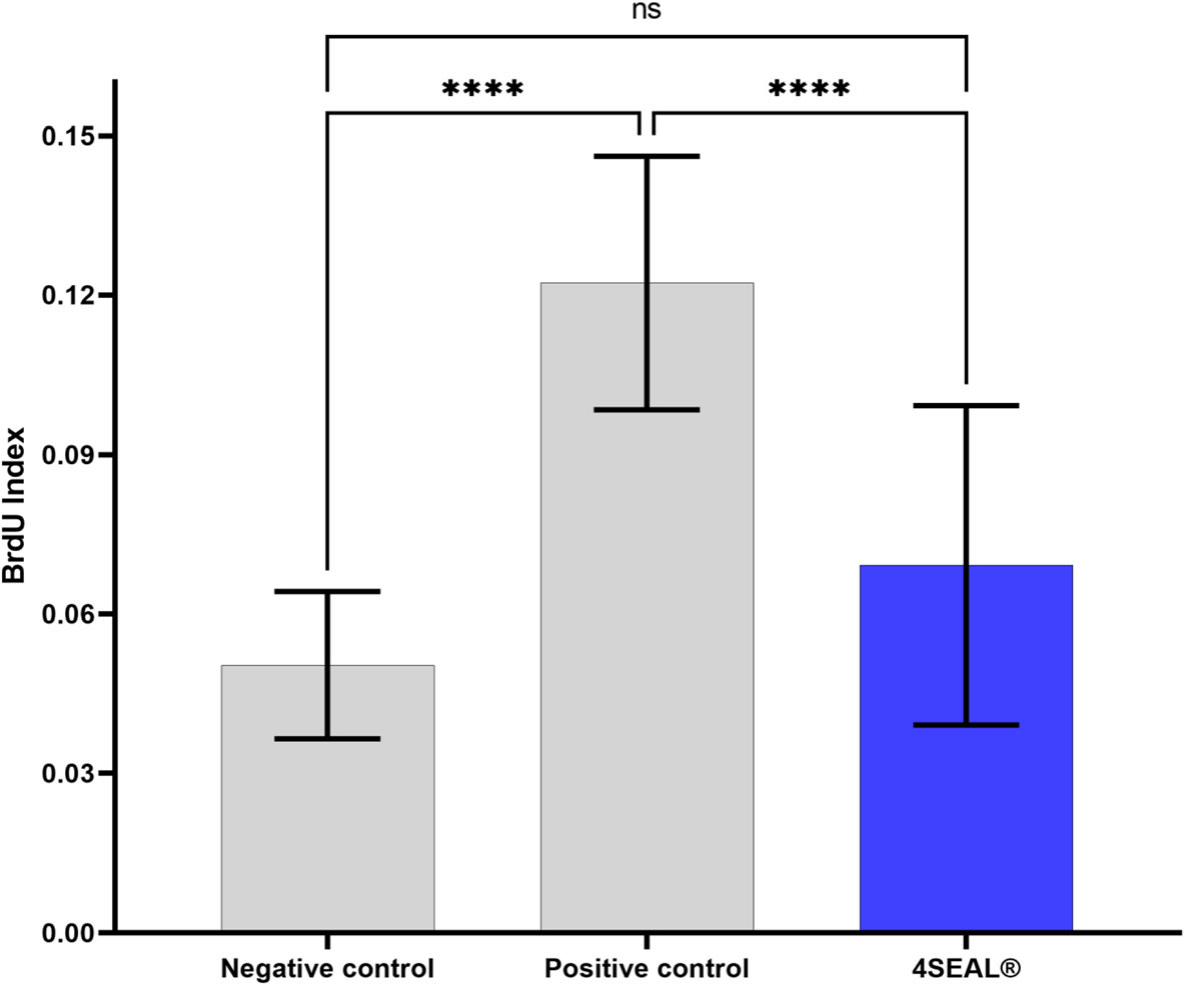
Absorbance results of the BrdU analysis of single-cell suspension of lymph node cells. No significant differences were observed between 4SEAL study group and negative control while statistical significance was observed between positive control and 4SEAL study group indicating no sensitization potential

None of the test or control animals exhibited overt signs of toxicity, enumerated in Annex C, Table C.1 – Common clinical signs and observations, ISO 10993 – 11, at any observation points. Furthermore, none of the animals treated with the test sample shows a significantly greater biological reactivity during the observation period than animals treated with vehicle control. None of the animals die, none of the animals’ lost 10% or more bodyweight. Animals showed minor signs of skin irritation – slight erythema was observed in all animals. Summarized results are presented in Table 17, Table 18, and Fig. 4.

**Table 17:** Change in body weight

Group	Average body weight change [%]
Negative control – acetone: olive oil (4:1 v/v)	−2.76
Extract of 4SEAL® – acetone: olive oil (4:1 v/v)	−0.40
Positive control - 25% HCA in acetone: olive oil (4:1 v/v)	1.31

**Table 18:** Absorbance results of the BrdU analysis

	Mean absorbance		BrdU Index	SI
	A450	A550		
PC	0.177	0.042	0.108	2.440
NC acetone: olive oil	0.106	0.044	0.033	N/A
4SEAL® acetone: olive oil	0.125	0.044	0.048	1.377
Blank	0.054	0.042	N/A	N/A

*NC* Negative control, *PC* Positive control

#### Intracutaneous reactivity

The Primary Irritation Index for sodium chloride and cottonseed oil extracts was calculated by subtracting the control group’s total Primary Irritation Score (PIS) from the total PIS of the study group. For sodium chloride and cottonseed oil extracts of 4SEAL® Hemostatic powder, the Primary Irritation Index was calculated to be 0.00. Results are presented in Table 19.

**Table 19:** Intracutaneous reactivity results

Group	24 h after injection		48 h after injection		72 h after injection		Total Primary Irritation Score
	Average Erythema	Average Oedema	Average Erythema	Average Oedema	Average Erythema	Average Oedema	
4SEAL® - Sodium Chloride	0	0	0	0	0	0	0
Solvent control - Sodium Chloride	0	0	0	0	0	0	0
4SEAL® - Cottonseed oil	0	0	0	0	0	0	0
Solvent control - Cottonseed oil	0	0	0	0	0	0	0

#### Acute systemic toxicity

None of the test or control animals exhibited overt signs of toxicity, enumerated in Annex C, Table C.1 – Common clinical signs and observations, ISO 10993 – 11, at any observation points [19]. Furthermore, none of the animals treated with the test sample shows a significantly greater biological reactivity during the observation period than animals treated with vehicle control. None of the animals die, none of the animals’ lost 10% or more bodyweight. Changes of bodyweight are presented in Table 20.

**Table 20:** Bodyweight changes

Group	Average body weight change 72 h after injection [%]
Solvent control -Sodium Chloride	0.95
4SEAL® - Sodium Chloride	−0.71
4SEAL® - Cottonseed Oil	6.07
Solvent control - Cottonseed Oil	3.51

#### Subacute toxicity combined with implantation

None of the test or control animals exhibited overt signs of toxicity, enumerated in Annex C, Table C.1 – Common clinical signs and observations, ISO 10993 – 11, at any observation points [19]. Furthermore, none of the animals treated with the test sample shows a significantly greater biological reactivity during the observation period than animals treated with vehicle control. None of the animals die, none of the animals’ lost 10% or more bodyweight. Bodyweight changes are presented in Table 21.

**Table 21:** Change in bodyweight

Group	Average body weight change after 28 days from exposition [%]	Average weight change after 28 days from exposition [g]
Negative control - female	14.50	30.92
4SEAL® - female	17.12	36.14
Negative control - male	24.19	75.06
4SEAL® - male	24.00	74.94

**Table 22:** Organ weight as a [%] of bodyweight

Group	Average body weight [g]	Average organ as a [%] of body weight							
		Brain	Heart	Lungs	Liver	Kidneys	Adrenal	Ovaries / testis	Spleen
Negative control - female	245.06	0.73	0.38	0.69	4.20	0.86	0.06	0.08	0.25
4SEAL® - female	247.02	0.78	0.33	0.81	4.20	0.82	0.06	0.11	0.27
Negative control - male	385.94	0.51	0.34	0.69	4.66	0.76	0.03	1.07	0.24
4SEAL® - male	388.58	0.53	0.34	0.73	4.82	0.75	0.04	1.00	0.22

##### Gross necropsy findings

No abnormalities have been found during gross necropsy. Places of subcutaneous implantation and the surrounding tissues did not show any abnormalities. During gross necropsy, organs (brain, lungs, heart, liver, kidneys, ovaries/testis, spleen) were weighted. Organ weight was divided by the animal’s body weight and is given as % of body weight. Test results are presented in Tables 22, 23, 24, 25, 26, 27, 28, 29, 30, 31, 32 and 33.

**Table 23:** Statistical comparison of the control group with the test group – *P*-value results

Sex	Brain	Heart	Lungs	Liver	Kidneys	Adrenal	Ovaries/testis	Spleen
female	0.02	0.30	0.37	0.98	0.27	1.00	0.08	0.32
male	0.40	0.89	0.59	0.06	0.75	0.59	0.12	0.11

**Table 24:** Biochemical finding results

Group	albumin	ALP	ALT	AST	Ca	Cl	Cholesterol	Creatinine
	[g/dL]	[U/L]	[U/L]	[U/L]	[mg/dL]	[mmol/L]	[mg/dL]	[mg/dL]
Negative control - female	4.12	257.00	38.80	154.80	9.46	102.20	69.00	0.21
4SEAL® - female	3.78	236.00	43.40	97.60	9.92	102.00	73.60	0.17
Negative control – male	3.36	245.80	44.20	81.40	10.28	98.40	73.00	0.20
4SEAL® - male	3.34	247.00	43.80	86.80	10.44	97.00	62.00	0.13

**Table 25:** Statistical comparison of the control group with the test group – P-value results

Sex	albumin	ALP	ALT	AST	Ca	Cl	Cholesterol	Creatinine
female	0.17	0.04	0.31	0.16	0.30	0.83	0.55	0.62
male	0.87	0.94	0.88	0.69	0.70	0.43	0.40	0.33

**Table 26:** Biochemical findings results

Group	GGT*	Glucose	K	P	Na	bilirubin	Total protein	Triglycerides	Blood urea nitrogen
	[U/L]	[mg/dL]	[mmol/L]	[mg/dL]	[mmol/L]	[mg/dL]	[g/dL]	[mg/dL]	[mg/dL]
Negative control - female	*	242.20	4.06	6.08	141.80	0.18	5.64	182.20	22.74
4SEAL® - female	*	247.20	4.44	6.30	141.00	0.24	5.48	101.40	18.88
Negative control – male	*	324.60	5.32	7.70	138.40	0.22	5.32	99.60	18.50
4SEAL® - male	*	333.60	5.14	7.72	136.00	0.18	5.28	146.00	26.50

*level undetected or very low

**Table 27:** Statistical comparison of the control group with the test group – P-value results

Sex	GGT	Glucose	K	P	Na	Bilirubin	Total protein	Triglycerides	Blood urea nitrogen
female	N/A	0.90	0.40	0.87	0.41	0.31	0.62	0.03	0.20
male	N/A	0.82	0.79	0.98	0.12	0.20	0.70	0.21	0.36

**Table 28:** Hematology findings

Group	PT	APTT	HGB	HCT	platelets	RBC’s	WBC	Lymphocytes	Monocytes	Granulocytes
	[sec.]	[sec.]	[g/dL]	[%]	[× 10 3/mm3]	[× 10 6/ mm3]	[× 10 3 /mm3]	[%]	[%]	[%]
Negative control – female	9.05	17.83	13.42	30.28	783.20	7.39	6.72	59.80	17.54	22.66
4SEAL® - female	7.80	20.08	12.80	29.30	752.40	7.23	5.40	53.50	17.36	29.02
Negative control – male	10.16	17.88	13.68	31.12	778.60	7.36	7.58	54.02	16.58	29.42
4SEAL® - male	11.28	22.08	13.16	30.16	748.80	7.39	6.14	55.24	17.88	26.88

**Table 29:** Statistical comparison of the control group with the test group – P-value

Sex	PT	APTT	HGB	HCT	platelets	RBC’s	WBC	Lymphocytes	Monocytes	Granulocytes
female	0.01	0.21	0.07	0.51	0.37	0.47	0.21	0.10	0.83	0.08
male	0.21	0.06	0.17	0.40	0.50	0.88	0.24	0.67	0.15	0.48

**Table 30:** Urine test results

Group	BLD	UBG	BIL	PRO	NIT	KET	GLU	pH	SG	LEU
	[Ery/μl]	[ml/dl]	[μmol/l]	[g/l]	[mg/dl]	[mg/dl]	[mg/dl]			[leu/μl]
Negative control - female	40.00	4.00	0.60	44.00	0.00	0.00	30.00	8.00	1.01	125.00
4SEAL® - female	12.00	4.80	1.00	38.00	0.00	0.00	0.00	7.60	1.02	105.00
Negative control – male	14.00	3.60	1.20	86.00	0.00	0.00	0.00	7.50	1.02	401.00
4SEAL® - male	12.00	6.40	1.40	100.00	0.00	15.00	0.00	8.00	1.01	405.00

**Table 31:** Statistical comparison of the control group with the test group – P-value

Sex	BLD	UBG	BIL	PRO	NIT	KET	GLU	pH	SG	LEU
female	0.08	0.66	0.52	0.79	N/A	N/A	0.37	0.37	0.51	0.89
male	0.89	0.10	0.81	0.37	N/A	0.07	N/A	0.30	0.46	0.98

**Table 32:** Histological evaluation of place implantation of study and control groups

	Negative control		Study group	
	Female	Male	Female	Male
**Cell type response**				
Polymorphonuclear cells	0	1	0	1
Lymphocytes	1	3	1	1
Plasma cells	0	1	0	0
Macrophages	3	4	3	3
Giant cells	1	2	0	1
Necrosis	0	1	0	0
**Subtotal (×2)**	10	24	8	12
**Tissue response**				
Neovascularization	3	2	3	3
Fibrosis	1	3	1	2
Subcutaneous changes	4	4	4	4
Fatty infiltrate	2	2	0	2
Muscular layer infiltration	3	2	0	3
**Subtotal**	23	49	16	32
**Total**	72		48	
**Average**	36		24

**Table 33:** Rating of reaction for implantation

Grade	Classification
0.0–2.9	Minimal or no reaction
3.0–8.9	Slight reaction
9.0–15.0	Moderate reaction
15.1	Severe reaction

Statistically significant differences were observed in brain weight between the control and test group of female rats. The microscopic and macroscopic observations did not show any abnormalities. The rest of the organs did not show statistically significant differences between the test and the control group.

Statistically significant differences were observed in ALP levels between the control and test group of female rats. However, the difference did not impact the clinical picture of the animals. Furthermore, there were no significant differences between the remaining parameters.

Statistically significant differences were observed in triglycerides and alkaline phosphatase levels between the control and test group of female rats. However, the difference did not impact the clinical picture of the animals. There were no significant differences between the remaining parameters.

Statistically significant differences were observed in prothrombin time between the control and test group of female rats. However, the difference did not impact the clinical picture of the animals. In addition, there were no significant differences between the remaining parameters.

No statistically significant differences were observed between the test and the control group.

According to ISO 10993-11, instead of full histopathology, limited analysis was conducted [19]. From each group, control, and study group, two representative animals were chosen. Following organs were examined: lungs, heart, liver, kidneys, ovaries/testis, spleen, bone, bone marrow. No abnormalities were found during histopathology evaluation. The microscopic structure of the organs was normal, with no signs of apoptosis of structural cells of individual organs. No significant differences between control and test groups of animals were detected.

As per ISO 10993-6, doubled cell-type response scores and tissue response scores were summarized and divided by the number of groups (male and female) to calculate the average score for test and control groups [18]. The final reaction rating was calculated by subtracting the average negative control score from the average tested sample score. The rating of reaction for 4SEAL® Hemostatic powder was −12 (0), which is classified as minimal or no reaction.

#### Pyrogenicity

At the end of the test, no rabbit showed an individual temperature rise higher or equal to 0.6 °C above its initial temperature. Pyrogenicity test results are presented in Table 34.

**Table 34:** Results of the pyrogenicity test

Rabbit No.	Rabbit weight [g]	Volume injected [mL]	Initial temperature [°C]	Maximal temperature [°C]	Temperature rise [°C]	Total temperature rise [°C]
1	3250	32.5	38.8	38.9	0.1	0.1
2	3970	39.7	38.75	38.7	−0.05	
3	3380	33.8	38.45	38.3	−0.15	

Summarized results of 4SEAL® Hemostatic powder biocompatibility testing are presented in Table 35 below.

**Table 35:** 4SEAL® Hemostatic powder biocompatibility testing summary

Test Performed	Extract(s)	Test and Control(s) Positive control (+) Negative control (−)	4SEAL® results
Chemical characterization ICP-MS ISO 10993-18 [8]	Water for injection	Water for injection (−)	Elements <LOD
Chemical characterization Headspace GC-MS ISO 10993-18 [8]	Raw product	Laboratory air (−) EPA VOC Mix 2 (+)	<AET
Chemical characterization GC-MS ISO 10993-18 [8]	Water for injection	Water for injection (−) Octane, decane, tridecane, tetradecane, hexadecane (+)	<AET
Cytotoxicity ISO 10993-5 [10]	MEM	Latex (+) HDPE (−)	No cytotoxicity
Sensitization ISO 10993-10 [17]	Acetone: olive oil	Acetone:olive oil (−) A-Hexylcinnamaldehyde (+)	No sensitization
Intracutaneous reactivity ISO 10993-10 [17]	Saline and CSO	Saline and CSO (−)	No irritation
Material mediated pyrogenicity ISO 10993-11 [19]	Saline	Saline (−)	No pyrogenicity
Endotoxin ISO 10993-11 [19]	Water for injection	Water for injection (−) Endotoxin standard (+)	< 20 EU/ 5 g device < 2.15 EU/ 3 g device
Acute systemic toxicity ISO 10993-11 [19]	Saline and CSO	Saline and CSO (−)	No signs of toxicity
Subacute toxicity combined with implantation ISO 10993-6 [18] ISO 10993-11 [19]	Direct implantation	Starsil Hemostat (−)	No signs of subacute toxicity No difference in tissue reaction
Genotoxicity (Ames) ISO 10993-3 [11]	Water for injection	Water for injection (−) without S9: 2-NF, 4-NQO, N4-ACT, 9-AA (+) with S9: 2-AA, 2-AF(+)	No mutagenic potential
Genotoxicity (MLA) ISO 10993-3 [11]	F5	F5(−) without S9: methylmethansulfonate (+) with S9: benzo [a] pyrene (+)	No mutagenic potential

## Discussion

The biocompatibility evaluation of medical devices is a complicated, multi-stage approach that aims to predict whether a medical device could present any potential danger in clinical use by evaluating the device’s compatibility with different biological systems. The process is regulated by internationally recognized standards such as the International Organization for Standardization (ISO) standard 10993 and a number of additional guidance from the FDA, Japanese Ministry of Health, Labor and Welfare, and other regulatory bodies [20]. The term medical device has a broad meaning and includes devices like a simple wooden spatula and highly complicated and sophisticated spine implant. Therefore, a different set of tests is needed for different categories of medical devices. 4SEAL® Hemostatic powder is degraded by alpha-amylases, glucoamylases, and macrophages in a few days. Therefore, 4SEAL® is classified as an implant that contacts tissue for a prolonged time (24 h to 30d) as per Table A.1 in ISO 10993-1 [6]. The tests performed in this study were chosen based on this classification.

The safety of novel 4SEAL® Hemostatic powder was investigated using quantitative and validated methods. The tested medical device proved to be fully biocompatible both in vivo and in vitro*. T*herefore, the study showed that the risk for 4SEAL® was not only mitigated, but the device is biologically safe. Furthermore, chemical testing employing Headspace GC-MS, GC-MS, and ICP-MS showed that there is no toxicological risk associated with the composition of 4SEAL® Hemostatic powder. The results from chemical testing combined with the data from mutagenicity and genotoxicity testing indicate with high probability that carcinogenesis risk associated with the use of 4SEAL® is negligible. The absence of substances of very high concern (SVHCs) and lack of genotoxic and mutagenic potential of 4SEAL® also shows that occurrence of late side effects is unlikely, and the use of this device is safe even in patients with genetic abnormalities.

What is more, the endotoxin content of the maximal size of the product (5 g) was 2.568 EU/device, which is well below the endotoxin limit for general medical devices. Results also indicate that the 3 g 4SEAL® Hemostatic powder may be used in procedures involving contact with cerebrospinal fluid for which the limit is 2.15 EU/device. This is of paramount importance as exposure to LPS leads to acute inflammation, impairment of amyloid-beta efflux, and disturbed CSF distribution [22, 23]. Finally, acute systemic toxicity testing provides information on health hazards likely to arise immediately after using the medical device, while subacute systemic toxicity testing provides data for an extended period of time. Often, the patients on which hemostatic devices are used sustained previous injuries in which case any subsequent stress on the body may have a significant effect on the patient’s heath. The results of in vivo studies showed no immediate or prolonged risk of toxicity associated with the use of 4SEAL®, indicating that it can be used regardless of the patient’s current condition.

## Conclusion

In conclusion, 4SEAL® Hemostatic powder showed excellent biocompatibility and should be considered safe for use. Therefore, 4SEAL® Hemostatic powder is a promising new hemostatic agent with a wide range of potential applications.

## Supplementary Information

**Additional file 1.**

## Data Availability

The following are available online at www.xxxxx.com/xxx/s1, Additional information and data for MLA, intracutaneous toxicity, sensitization studies, acute systemic toxicity, subacute systemic toxicity with implantation and pyrogenicity are presented in Tables S1-S15 and Fig. S1.

## Abbreviations

AET: Analytical Evaluation Threshold
AMES: Bacterial Reverse Mutation Test
LLNA: Local Lymph Node Assay
LNC: Lymph Node Cells
MLA: Mouse Lymphoma Assay
PC: Positive Control
RPE: Relative Plating Efficiency
RSG: Relative Suspension Growth
RTG: Relative Total Growth
SI: Sensitization Index
SVOC: Semi-Volatile Organic Compound
TEE: Total Element Exposure
VOC: Volatile Organic Compound
