# Supplementary material for: ISO 10993 biological evaluation of novel hemostatic powder – 4SEAL®
Source: Biomater Res. 2022 Apr 5;26:12. doi: 10.1186/s40824-022-00258-6 (PMC8981750; doi:10.1186/s40824-022-00258-6)

**Additional information for MLA study.**

Table S1. MLA acceptance criteria

| **Negative control** | |
| --- | --- |
| Mutant frequency (MF) of negative control | 35 to 140 TFT resistant mutants per 10^6^ cells |
| Plating efficiency of the negative control | between 60% and 120% |
| Total suspension growth (TSG) | 8-32 for 4h treatment and 32-180 for 24h treatment |
| **Positive control** | |
| 4h treatment | ≥ 100 mutants over negative control  or  small colony MF ≥ 300 (*10-6) over negative control |
| 24h treatment | MF ≥ 300 (*10-6) over negative control with 40% small colonies  or  small colony MF ≥ 300 (*10-6) over negative control |

Table S2. Reliability check of the assay.

| Conditions | Mutant frequency of NC | Cloning efficiency of NC | Total suspension growth of NC | Positive control |
| --- | --- | --- | --- | --- |
| 4h exposure, without metabolic activation | Met criteria | Met criteria | Met criteria | Met criteria |
| 4h exposure, with metabolic activation | Met criteria | Met criteria | Met criteria | Met criteria |
| 24h exposure, without metabolic activation | Met criteria | Met criteria | Met criteria | Met criteria |

**Media compositions**

F5 medium

RPMI 1640 medium supplemented with 5% horse serum, 1% penicillin/streptomycin, 1% Pluronic F-68, and 1% sodium pyruvate.

F10 medium

RPMI 1640 medium supplemented with 10% horse serum, 1% penicillin/streptomycin, 1% Pluronic F-68, and 1% sodium pyruvate.

F20 medium

RPMI 1640 medium supplemented with 20% horse serum, 1% penicillin/streptomycin, 1% Pluronic F-68, and 1% sodium pyruvate.

Selective F20 medium **with TFT**

RPMI 1640 medium supplemented with 20% horse serum, 1% penicillin/streptomycin, 1% Pluronic F-68, 1% sodium pyruvate, and 3 μg/ml TFT.

THMG 100x stock

300 μg/ml Thymidine

500 μg/ml Hypoxanthine

750 μg/ml Glycine

10 μg/ml Methotrexate

THG 100x stock

300 μg/ml Thymidine

500 μg/ml Hypoxanthine

750 μg/ml Glycine

S9 mix (per sample)

S9 fraction 0.1 ml

150 mM KCl 0.1 ml

638 mM G6P 0.1 ml

33 mM NADP 0.1 ml

dH2O 0.1 ml

pH adjusted to 7 with 1 N NaOH.

For each 4h treatment with presence of S9 fraction 0.5 ml of S9 mix per 9.5 ml of cell culture medium was used resulting in the final concentration of S9 fraction at 1%.

Negative control: F5 medium with or without S9 fraction.

Positive controls: 10 μg/ml methylmethansulfonate for cultures without S9 fraction

3 μg/ml benzo[a]pyrene for cultures with S9 fraction

**Detailed data for Intracutaneous Toxicity**

Table S3. Polar vehicle results.

| **EXTRACT: SODIUM CHLORIDE INJECTION** | | | | | | | | | |
| --- | --- | --- | --- | --- | --- | --- | --- | --- | --- |
| Rabbit No. | Site N. | 24 hour after injection | | 48 hour after injection | | 72 hour after injection | | PIS | PIS  total |
|  |  | **E** | **O** | **E** | **O** | **E** | **O** |  |  |
| 7 | 1 | 0 | 0 | 0 | 0 | 0 | 0 | 0 | 0 |
|  | 2 | 0 | 0 | 0 | 0 | 0 | 0 |  |  |
|  | 3 | 0 | 0 | 0 | 0 | 0 | 0 |  |  |
|  | 4 | 0 | 0 | 0 | 0 | 0 | 0 |  |  |
|  | 5 | 0 | 0 | 0 | 0 | 0 | 0 |  |  |
| 9 | 1 | 0 | 0 | 0 | 0 | 0 | 0 | 0 |  |
|  | 2 | 0 | 0 | 0 | 0 | 0 | 0 |  |  |
|  | 3 | 0 | 0 | 0 | 0 | 0 | 0 |  |  |
|  | 4 | 0 | 0 | 0 | 0 | 0 | 0 |  |  |
|  | 5 | 0 | 0 | 0 | 0 | 0 | 0 |  |  |
| 10 | 1 | 0 | 0 | 0 | 0 | 0 | 0 | 0 |  |
|  | 2 | 0 | 0 | 0 | 0 | 0 | 0 |  |  |
|  | 3 | 0 | 0 | 0 | 0 | 0 | 0 |  |  |
|  | 4 | 0 | 0 | 0 | 0 | 0 | 0 |  |  |
|  | 5 | 0 | 0 | 0 | 0 | 0 | 0 |  |  |
| **SOLVENT CONTROL: SODIUM CHLORIDE INJECTION** | | | | | | | | | |
| Rabbit N. | Site N. | 24 hour after injection | | 48 hour after injection | | 72 hour after injection | | PIS | PIS  total |
|  |  | E | O | E | O | E | O |  |  |
| 7 | 1 | 0 | 0 | 0 | 0 | 0 | 0 | 0 | 0 |
|  | 2 | 0 | 0 | 0 | 0 | 0 | 0 |  |  |
|  | 3 | 0 | 0 | 0 | 0 | 0 | 0 |  |  |
|  | 4 | 0 | 0 | 0 | 0 | 0 | 0 |  |  |
|  | 5 | 0 | 0 | 0 | 0 | 0 | 0 |  |  |
| 9 | 1 | 0 | 0 | 0 | 0 | 0 | 0 | 0 |  |
|  | 2 | 0 | 0 | 0 | 0 | 0 | 0 |  |  |
|  | 3 | 0 | 0 | 0 | 0 | 0 | 0 |  |  |
|  | 4 | 0 | 0 | 0 | 0 | 0 | 0 |  |  |
|  | 5 | 0 | 0 | 0 | 0 | 0 | 0 |  |  |
| 10 | 1 | 0 | 0 | 0 | 0 | 0 | 0 | 0 |  |
|  | 2 | 0 | 0 | 0 | 0 | 0 | 0 |  |  |
|  | 3 | 0 | 0 | 0 | 0 | 0 | 0 |  |  |
|  | 4 | 0 | 0 | 0 | 0 | 0 | 0 |  |  |
|  | 5 | 0 | 0 | 0 | 0 | 0 | 0 |  |  |

E = Erythema O = Oedema PIS = Primary Irritation Score

PRIMARY IRRITARION INDEX in Sodium Chloride injection (TREATED – CONTROL): 0.00

Table S4. Non-polar vehicle results.

| **EXTRACT IN COTTONSEED OIL** | | | | | | | | | |
| --- | --- | --- | --- | --- | --- | --- | --- | --- | --- |
| Rabbit No. | Site N. | 24 hour after injection | | 48 hour after injection | | 72 hour after injection | | PIS | PIS  total |
|  |  | E | O | E | O | E | O |  |  |
| 7 | 1 | 0 | 0 | 0 | 0 | 0 | 0 | 0 | 0 |
|  | 2 | 0 | 0 | 0 | 0 | 0 | 0 |  |  |
|  | 3 | 0 | 0 | 0 | 0 | 0 | 0 |  |  |
|  | 4 | 0 | 0 | 0 | 0 | 0 | 0 |  |  |
|  | 5 | 0 | 0 | 0 | 0 | 0 | 0 |  |  |
| 9 | 1 | 0 | 0 | 0 | 0 | 0 | 0 | 0 |  |
|  | 2 | 0 | 0 | 0 | 0 | 0 | 0 |  |  |
|  | 3 | 0 | 0 | 0 | 0 | 0 | 0 |  |  |
|  | 4 | 0 | 0 | 0 | 0 | 0 | 0 |  |  |
|  | 5 | 0 | 0 | 0 | 0 | 0 | 0 |  |  |
| 10 | 1 | 0 | 0 | 0 | 0 | 0 | 0 | 0 |  |
|  | 2 | 0 | 0 | 0 | 0 | 0 | 0 |  |  |
|  | 3 | 0 | 0 | 0 | 0 | 0 | 0 |  |  |
|  | 4 | 0 | 0 | 0 | 0 | 0 | 0 |  |  |
|  | 5 | 0 | 0 | 0 | 0 | 0 | 0 |  |  |
| **SOLVENT CONTROL: COTTONSEED OIL** | | | | | | | | | |
| Rabbit N. | Site N. | 24 hour after injection | | 48 hour after injection | | 72 hour after injection | | PIS | PIS  total |
|  |  | E | O | E | O | E | O |  |  |
| 7 | 1 | 0 | 0 | 0 | 0 | 0 | 0 | 0 | 0 |
|  | 2 | 0 | 0 | 0 | 0 | 0 | 0 |  |  |
|  | 3 | 0 | 0 | 0 | 0 | 0 | 0 |  |  |
|  | 4 | 0 | 0 | 0 | 0 | 0 | 0 |  |  |
|  | 5 | 0 | 0 | 0 | 0 | 0 | 0 |  |  |
| 9 | 1 | 0 | 0 | 0 | 0 | 0 | 0 | 0 |  |
|  | 2 | 0 | 0 | 0 | 0 | 0 | 0 |  |  |
|  | 3 | 0 | 0 | 0 | 0 | 0 | 0 |  |  |
|  | 4 | 0 | 0 | 0 | 0 | 0 | 0 |  |  |
|  | 5 | 0 | 0 | 0 | 0 | 0 | 0 |  |  |
| 10 | 1 | 0 | 0 | 0 | 0 | 0 | 0 | 0 |  |
|  | 2 | 0 | 0 | 0 | 0 | 0 | 0 |  |  |
|  | 3 | 0 | 0 | 0 | 0 | 0 | 0 |  |  |
|  | 4 | 0 | 0 | 0 | 0 | 0 | 0 |  |  |
|  | 5 | 0 | 0 | 0 | 0 | 0 | 0 |  |  |

E = Erythema O = Oedema PIS = Primary Irritation Score

PRIMARY IRRITARION INDEX in Cottonseed Oil (TREATED – CONTROL): 0.00

**Detailed data for Sensitization studies**

Table S5. Body weight – sensitization studies.

| **Animal No.** |  | **Body weight (g)** | | **Body weight change [%]** |
| --- | --- | --- | --- | --- |
|  |  | **Beginning of the test** | **End of the test** |  |
| **225** | Solvent control – acetone: olive oil (4:1 v/v) | 19.5 | 19.1 | -3.05% |
| **226** |  | 21 | 21.6 | -3.14% |
| **227** |  | 18.9 | 19 | -1.04% |
| **228** |  | 20.7 | 20.6 | -3.74% |
| **229** |  | 20.3 | 20.6 | -2.83% |
| **230** | Extract of control article – acetone: olive oil (4:1 v/v) | 19.8 | 20 | -1.48% |
| **231** |  | 19.9 | 20.6 | 3.52% |
| **232** |  | 19.2 | 19.1 | -2.05% |
| **233** |  | 20.5 | 20 | -0.50% |
| **234** |  | 19.7 | 19.6 | -1.51% |
| **235** | Positive control - 25% HCA in acetone: olive oil (4:1 v/v) | 19.1 | 19 | 1.60% |
| **236** |  | 19.7 | 20.4 | 0.49% |
| **237** |  | 19.6 | 19.5 | 2.09% |
| **238** |  | 19.1 | 19.1 | 1.06% |
| **239** |  | 19.5 | 18.8 | -3.09% |

**Detailed data for Acute Systemic Toxicity Studies**

Table S6. Body weight – acute systemic toxicity studies.

|  | **Mouse No.** | **Body weight [g]** | | | | **Body weight change [%]** |
| --- | --- | --- | --- | --- | --- | --- |
|  |  | **Before dosing** | **24 h after injection** | **48 h after injection** | **72 h after injection** |  |
| **SOLVENT CONTROL -SODIUM CHLORIDE** | **240** | 19 | 19.3 | 19.2 | 19.5 | 2.63% |
|  | **241** | 18.8 | 19.3 | 18.8 | 19.1 | 1.60% |
|  | **242** | 18.6 | 18.4 | 18.2 | 18.6 | 0.00% |
|  | **243** | 19.6 | 19.7 | 19.3 | 19.7 | 0.51% |
|  | **244** | 18.6 | 18.3 | 17.9 | 18.6 | 0.00% |
| **EXTRACT -SODIUM CHLORIDE** | **205** | 20.3 | 20 | 20 | 19.9 | -1.97% |
|  | **206** | 20.7 | 21.2 | 21.2 | 20.9 | 0.97% |
|  | **207** | 22 | 21.6 | 21.2 | 21.2 | -3.64% |
|  | **208** | 20 | 20 | 20 | 20 | 0.00% |
|  | **209** | 18.6 | 18.5 | 18.6 | 18.8 | 1.08% |
| **EXTRACT - COTTONSEED OIL** | **215** | 20.6 | 21.2 | 21.5 | 22.2 | 7.77% |
|  | **216** | 21.4 | 22.2 | 22.7 | 22.6 | 5.61% |
|  | **217** | 21.5 | 21.8 | 22.3 | 22.9 | 6.51% |
|  | **218** | 18.3 | 19.3 | 19.8 | 19.8 | 8.20% |
|  | **219** | 22 | 21.9 | 22.4 | 22.5 | 2.27% |
| **SOLVENT CONTROL - COTTONSEED OIL** | **220** | 20.3 | 20.5 | 20.3 | 21 | 3.45% |
|  | **221** | 20.5 | 21.5 | 20.9 | 21.2 | 3.41% |
|  | **222** | 22.6 | 23.1 | 23.3 | 23.5 | 3.98% |
|  | **223** | 21.4 | 21.6 | 21.5 | 21.9 | 2.34% |
|  | **224** | 20.6 | 20.7 | 20.8 | 21.5 | 4.37% |

**Detailed data for Subacute systemic toxicity with implantation**

Table S7. Body weight – subacute systemic toxicity studies.

| **Animal No.** | **Sample** | **Body weight [g]** | | | | | | | | **Body weight change [%]** | **Weight change [g]** |
| --- | --- | --- | --- | --- | --- | --- | --- | --- | --- | --- | --- |
|  |  | **Day of the test** | | | | | | | |  |  |
|  |  | **1** | **2** | **3** | **4** | **7** | **14** | **21** | **28** |  |  |
| 121 | Negative control - female | 215 | 209.6 | 216.3 | 213.1 | 219.7 | 223 | 235.8 | 242.2 | 12.65% | 27.2 |
| 122 |  | 224.5 | 220.7 | 224 | 228 | 226.9 | 230 | 236.5 | 250.5 | 11.58% | 26 |
| 123 |  | 214.3 | 211.5 | 211.4 | 216 | 216.2 | 225 | 236.1 | 244 | 13.86% | 29.7 |
| 124 |  | 207.2 | 203 | 206.2 | 203.3 | 212 | 220 | 230.9 | 246 | 18.73% | 38.8 |
| 125 |  | 209.7 | 205.7 | 210 | 211.7 | 217 | 226 | 226.6 | 242.6 | 15.69% | 32.9 |
| 126 | Study group - female | 215.5 | 218.2 | 219.7 | 219.8 | 225 | 232 | 235.8 | 252.7 | 17.26% | 37.2 |
| 127 |  | 216.5 | 217.5 | 211.4 | 211.2 | 224.8 | 233.3 | 238.5 | 253.2 | 16.95% | 36.7 |
| 128 |  | 203.5 | 207.9 | 208 | 212.8 | 210.2 | 220 | 223.6 | 235.4 | 15.68% | 31.9 |
| 129 |  | 205.2 | 204.5 | 208 | 210 | 215.8 | 224 | 230.2 | 239.8 | 16.86% | 34.6 |
| 130 |  | 213.7 | 213.4 | 214.2 | 216 | 220.5 | 236 | 240.3 | 254 | 18.86% | 40.3 |
| 131 | Negative control - male | 298.3 | 297.2 | 298.2 | 303 | 320 | 335 | 348.9 | 373.9 | 25.34% | 75.6 |
| 132 |  | 315.6 | 317.8 | 322 | 322.2 | 346 | 360 | 374.9 | 388.5 | 23.10% | 72.9 |
| 133 |  | 311.7 | 310.7 | 321.2 | 315.8 | 336.5 | 346 | 362.1 | 375 | 20.31% | 63.3 |
| 134 |  | 307.2 | 309.1 | 314.6 | 317.5 | 342.8 | 357 | 375.5 | 399.5 | 30.05% | 92.3 |
| 135 |  | 321.6 | 316.5 | 321 | 325.2 | 345.9 | 359 | 375.8 | 392.8 | 22.14% | 71.2 |
| 136 | Study group - male | 297.8 | 297.8 | 305.8 | 308 | 326.1 | 340 | 349.9 | 358.8 | 20.48% | 61 |
| 137 |  | 326.5 | 323 | 332.8 | 333 | 353.3 | 368 | 385.3 | 411 | 25.88% | 84.5 |
| 138 |  | 309 | 307 | 313 | 314 | 333 | 344 | 362.6 | 389.1 | 25.92% | 80.1 |
| 139 |  | 313.7 | 310.5 | 310.7 | 312.3 | 337.1 | 354 | 376.6 | 394.8 | 26.10% | 81.1 |
| 140 |  | 321.2 | 315.2 | 314.4 | 317 | 336 | 345 | 361.6 | 389.2 | 21.63% | 68 |

Table S8. Organ weight – subacute systemic toxicity studies.

| **Animal No.** | **Sample** | **Body weight [g]** | **Organ weight [%]** | | | | | | | |
| --- | --- | --- | --- | --- | --- | --- | --- | --- | --- | --- |
|  |  |  | **Brain** | **Heart** | **Lungs** | **Liver** | **Kidneys** | **Adrenal** | **Ovaries/testis** | **Spleen** |
| 121 | Negative control - female | 242.2 | 0.70 | 0.33 | 0.66 | 4.29 | 0.87 | 0.04 | 0.08 | 0.25 |
| 122 |  | 250.5 | 0.72 | 0.36 | 0.68 | 4.19 | 0.88 | 0.08 | 0.08 | 0.24 |
| 123 |  | 244 | 0.70 | 0.33 | 0.61 | 3.89 | 0.98 | 0.08 | 0.08 | 0.25 |
| 124 |  | 246 | 0.77 | 0.49 | 0.73 | 4.23 | 0.81 | 0.04 | 0.08 | 0.24 |
| 125 |  | 242.6 | 0.74 | 0.37 | 0.78 | 4.41 | 0.78 | 0.04 | 0.08 | 0.25 |
| 126 | Study group - female | 252.7 | 0.75 | 0.24 | 0.71 | 4.12 | 0.79 | 0.08 | 0.12 | 0.24 |
| 127 |  | 253.2 | 0.79 | 0.36 | 0.63 | 4.50 | 0.87 | 0.04 | 0.08 | 0.24 |
| 128 |  | 235.4 | 0.81 | 0.34 | 0.64 | 4.16 | 0.85 | 0.08 | 0.13 | 0.34 |
| 129 |  | 239.8 | 0.75 | 0.33 | 1.25 | 4.13 | 0.79 | 0.04 | 0.08 | 0.29 |
| 130 |  | 254 | 0.79 | 0.39 | 0.83 | 4.09 | 0.79 | 0.04 | 0.12 | 0.24 |
| 131 | Negative control - male | 373.9 | 0.51 | 0.37 | 0.72 | 4.60 | 0.75 | 0.05 | 1.12 | 0.27 |
| 132 |  | 388.5 | 0.51 | 0.31 | 0.62 | 4.76 | 0.75 | 0.03 | 1.08 | 0.23 |
| 133 |  | 375 | 0.53 | 0.37 | 0.67 | 4.61 | 0.77 | 0.03 | 1.01 | 0.24 |
| 134 |  | 399.5 | 0.53 | 0.30 | 0.68 | 4.66 | 0.78 | 0.03 | 1.00 | 0.25 |
| 135 |  | 392.8 | 0.48 | 0.33 | 0.74 | 4.68 | 0.76 | 0.03 | 1.12 | 0.23 |
| 136 | Study group - male | 358.8 | 0.53 | 0.42 | 0.95 | 4.63 | 0.78 | 0.03 | 1.00 | 0.22 |
| 137 |  | 411 | 0.51 | 0.32 | 0.54 | 4.99 | 0.75 | 0.02 | 1.02 | 0.24 |
| 138 |  | 389.1 | 0.57 | 0.33 | 0.72 | 4.88 | 0.82 | 0.05 | 1.00 | 0.23 |
| 139 |  | 394.8 | 0.51 | 0.30 | 0.63 | 4.86 | 0.66 | 0.05 | 0.91 | 0.20 |
| 140 |  | 389.2 | 0.51 | 0.33 | 0.80 | 4.75 | 0.75 | 0.03 | 1.08 | 0.23 |

Table S9. Biochemical findings – subacute systemic toxicity studies.

| **Animal No.** | **Sample** | **albumin** | **ALP** | **ALT** | **AST** | **Ca** | **Cl** | **Cholesterol** | **Creatinine** |
| --- | --- | --- | --- | --- | --- | --- | --- | --- | --- |
|  |  | [g/dL] | [U/L] | [U/L] | [U/L] | [mg/dL] | [mmol/L] | [mg/dL] | [mg/dL] |
| 121 | Negative control - female | 4.3 | 246 | 47 | 211 | 9.5 | 102 | 89 | 0.39 |
| 122 |  | 3.6 | 258 | 28 | 189 | 9.5 | 101 | 62 | 0.01 |
| 123 |  | 3.9 | 257 | 33 | 124 | 10.3 | 102 | 57 | 0.21 |
| 124 |  | 4.8 | 254 | 40 | 192 | 8.9 | 105 | 59 | 0.24 |
| 125 |  | 4 | 270 | 46 | 58 | 9.1 | 101 | 78 | 0.21 |
| 126 | Study group - female | 3.8 | 235 | 40 | 167 | 8.7 | 103 | 71 | 0.01 |
| 127 |  | 3.7 | 215 | 43 | 49 | 10.4 | 100 | 77 | 0.19 |
| 128 |  | 3.8 | 245 | 42 | 67 | 10.6 | 103 | 65 | 0.24 |
| 129 |  | 4 | 256 | 41 | 70 | 10.2 | 102 | 86 | 0.2 |
| 130 |  | 3.6 | 229 | 51 | 135 | 9.7 | 102 | 69 | 0.23 |
| 131 | Negative control - male | 3.2 | 224 | 47 | 69 | 9.6 | 103 | 70 | 0.01 |
| 132 |  | 3.3 | 266 | 39 | 73 | 10.4 | 99 | 69 | 0.22 |
| 133 |  | 3.6 | 244 | 52 | 94 | 10.3 | 96 | 75 | 0.25 |
| 134 |  | 3.4 | 274 | 44 | 76 | 10.8 | 100 | 79 | 0.3 |
| 135 |  | 3.3 | 221 | 39 | 95 | 10.3 | 94 | 72 | 0.21 |
| 136 | Study group - male | 3.7 | 240 | 42 | 66 | 10.9 | 96 | 61 | 0.22 |
| 137 |  | 3.3 | 206 | 44 | 76 | 9.2 | 98 | 78 | 0.21 |
| 138 |  | 3.2 | 266 | 45 | 66 | 10.8 | 97 | 83 | 0.18 |
| 139 |  | 3.3 | 277 | 44 | 127 | 10.2 | 96 | 18 | 0.01 |
| 140 |  | 3.2 | 246 | 44 | 99 | 11.1 | 98 | 70 | 0.01 |

Table S10. Biochemical findings – subacute systemic toxicity studies.

| **Animal No.** | **Sample** | **GGT*** | **Glucose** | **K** | **P** | **Na** | **bilirubin** | **Total protein** | **Triglycerides** | **Blood urea nitrogen** |
| --- | --- | --- | --- | --- | --- | --- | --- | --- | --- | --- |
|  |  | [U/L] | [mg/dL] | [mmol/L] | [mg/dL] | [mmol/L] | [mg/dL] | [g/dL] | [mg/dL] | [mg/dL] |
| 121 | Negative control - female | * | 177 | 4.4 | 5.4 | 142 | 0.1 | 6.5 | 170 | 29 |
| 122 |  | * | 196 | 4.2 | 3.4 | 142 | 0.2 | 5.3 | 236 | 28 |
| 123 |  | * | 227 | 4.1 | 5.9 | 139 | 0.3 | 5.7 | 163 | 19.9 |
| 124 |  | * | 372 | 4.4 | 10.1 | 143 | 0.1 | 4.8 | 104 | 17.2 |
| 125 |  | * | 239 | 3.2 | 5.6 | 143 | 0.2 | 5.9 | 238 | 19.6 |
| 126 | Study group - female | * | 266 | 4.7 | 7.8 | 141 | 0.4 | 5.7 | 108 | 22.1 |
| 127 |  | * | 273 | 3.9 | 6.1 | 140 | 0.2 | 5.4 | 86 | 16.7 |
| 128 |  | * | 225 | 5.6 | 8.2 | 140 | 0.2 | 5.7 | 57 | 18.5 |
| 129 |  | * | 256 | 3.5 | 5 | 143 | 0.2 | 5.3 | 99 | 20.3 |
| 130 |  | * | 216 | 4.5 | 4.4 | 141 | 0.2 | 5.3 | 157 | 16.8 |
| 131 | Negative control - male | * | 249 | 4.4 | 6.1 | 141 | 0.2 | 5.3 | 123 | 21.4 |
| 132 |  | * | 357 | 5.7 | 7.9 | 139 | 0.2 | 5.2 | 86 | 19.5 |
| 133 |  | * | 323 | 5.7 | 7.8 | 138 | 0.2 | 5.5 | 115 | 17.8 |
| 134 |  | * | 414 | 6.4 | 8.9 | 137 | 0.3 | 5.3 | 105 | 17.4 |
| 135 |  | * | 280 | 4.4 | 7.8 | 137 | 0.2 | 5.3 | 69 | 16.4 |
| 136 | Study group - male | * | 384 | 6.2 | 9.2 | 136 | 0.2 | 5.4 | 75 | 21.4 |
| 137 |  | * | 255 | 4.2 | 6.5 | 134 | 0.2 | 5.5 | 110 | 18.3 |
| 138 |  | * | 297 | 4.2 | 6.1 | 133 | 0.2 | 5.2 | 139 | 15.9 |
| 139 |  | * | 369 | 4.6 | 7.6 | 139 | 0.1 | 5.3 | 258 | 57 |
| 140 |  | * | 363 | 6.5 | 9.2 | 138 | 0.2 | 5 | 148 | 19.9 |

*level undetected or very low

Table S11. Hematology findings – subacute systemic toxicity studies.

| **Animal No.** | **Sample** | **PT** | **APTT** | **HGB** | **HCT** | **platelets** | **RBC's** | **WBC** | **Lymphocytes** | **Monocytes** | **Granulocytes** |
| --- | --- | --- | --- | --- | --- | --- | --- | --- | --- | --- | --- |
|  |  | [sec.] | [sec.] | [g/dL] | [%] | [x 10 3/mm3] | [x 10 6/ mm3] | [x 10 3 /mm3] | [%] | [%] | [%] |
| 121 | Negative control - female | - | - | 12.8 | 28.2 | 788 | 6.82 | 5.8 | 59.50 | 18.20 | 22.30 |
| 122 |  | 9 | 19.4 | 13.4 | 29.4 | 871 | 7.15 | 4.2 | 60.10 | 16.20 | 23.70 |
| 123 |  | 8.9 | 14.7 | 13.6 | 29.9 | 805 | 7.37 | 6.9 | 63.80 | 15.70 | 20.50 |
| 124 |  | 9 | 19.3 | 13.6 | 32.2 | 739 | 7.78 | 8.3 | 58.20 | 18.50 | 23.30 |
| 125 |  | 9.3 | 17.9 | 13.7 | 31.7 | 713 | 7.84 | 8.4 | 57.40 | 19.10 | 23.50 |
| 126 | Study group - female | 7.1 | 20.6 | 12.8 | 27.7 | 806 | 7.5 | 7.2 | 51.80 | 18.20 | 30.00 |
| 127 |  | 7.5 | 19.7 | 12.6 | 27.6 | 759 | 7.08 | 4.6 | 48.10 | 17.10 | 34.20 |
| 128 |  | 7.6 | 19.4 | 12.5 | 27.1 | 707 | 7.01 | 4.1 | 49.30 | 16.80 | 33.90 |
| 129 |  | 8.5 | 16.6 | 12.4 | 33.4 | 751 | 7.38 | 5.4 | 64.60 | 16.20 | 19.20 |
| 130 |  | 8.3 | 24.1 | 13.7 | 30.7 | 739 | 7.17 | 5.7 | 53.70 | 18.50 | 27.80 |
| 131 | Negative control - male | 8.6 | 18.7 | 14 | 32.2 | 779 | 7.44 | 9.4 | 52.00 | 16.80 | 31.20 |
| 132 |  | 8.2 | 15 | 13.7 | 30.4 | 849 | 7.66 | 9.8 | 55.70 | 16.40 | 27.90 |
| 133 |  | 12.1 | 17.3 | 14 | 33.2 | 654 | 7.46 | 5 | 47.00 | 14.50 | 38.50 |
| 134 |  | 11.2 | 17.8 | 12.8 | 28.9 | 780 | 6.87 | 5.9 | 59.60 | 18.60 | 21.90 |
| 135 |  | 10.7 | 20.6 | 13.9 | 30.9 | 831 | 7.37 | 7.8 | 55.80 | 16.60 | 27.60 |
| 136 | Study group - male | 11.4 | 24.1 | 13.6 | 30.3 | 800 | 7.39 | 6.7 | 60.60 | 17.50 | 21.90 |
| 137 |  | 11.4 | 24 | 12.9 | 32.1 | 714 | 7.46 | 6.8 | 57.10 | 19.80 | 23.10 |
| 138 |  | 11.3 | 25 | 12.9 | 29 | 807 | 7.72 | 7.4 | 54.00 | 17.50 | 28.50 |
| 139 |  | 11.1 | 20.9 | 13.9 | 31.6 | 746 | 7.28 | 5.8 | 54.60 | 17.50 | 27.90 |
| 140 |  | 11.2 | 16.4 | 12.5 | 27.8 | 677 | 7.08 | 4 | 49.90 | 17.10 | 33.00 |

Table S12. Urine test results – subacute systemic toxicity studies.

| **Animal No.** | **Sample** | **BLD** | **UBG** | **BIL** | **PRO** | **NIT** | **KET** | **GLU** | **pH** | **SG** | **LEU** |
| --- | --- | --- | --- | --- | --- | --- | --- | --- | --- | --- | --- |
|  |  | [Ery/µl] | [ml/dl] | [µmol/l] | [g/l] | [mg/dl] | [mg/dl] | [mg/dl] |  |  | [leu/µl] |
| 121 | Negative control - female | 0 | 4 | 1 | 100 | 0 | 0 | 0 | 8 | 1.010 | 500 |
| 122 |  | 50 | 2 | 0 | 30 | 0 | 0 | 0 | 8 | 1.015 | 75 |
| 123 |  | 50 | 2 | 0 | 30 | 0 | 0 | 150 | 8 | 1.015 | 0 |
| 124 |  | 50 | 8 | 2 | 30 | 0 | 0 | 0 | 8 | 1.010 | 25 |
| 125 |  | 50 | 4 | 0 | 30 | 0 | 0 | 0 | 8 | 1.015 | 25 |
| 126 | Study group - female | 0 | 2 | 0 | 30 | 0 | 0 | 0 | 7 | 1.030 | 0 |
| 127 |  | 0 | 8 | 1 | 100 | 0 | 0 | 0 | 7 | 1.030 | 0 |
| 128 |  | 50 | 2 | 0 | 30 | 0 | 0 | 0 | 7 | 1.010 | 500 |
| 129 |  | 0 | 8 | 2 | 30 | 0 | 0 | 0 | 9 | 1.010 | 25 |
| 130 |  | 10 | 4 | 2 | 0 | 0 | 0 | 0 | 8 | 1.005 | 0 |
| 131 | Negative control - male | 50 | 2 | 0 | 100 | 0 | 0 | 0 | 8 | 1.015 | 500 |
| 132 |  | 0 | 2 | 0 | 30 | 0 | 0 | 0 | 7 | 1.015 | 5 |
| 133 |  | 0 | 2 | 1 | 100 | 0 | 0 | 0 | 8 | 1.010 | 500 |
| 134 |  | 10 | 8 | 4 | 100 | 0 | 0 | 0 | 8 | 1.020 | 500 |
| 135 |  | 10 | 4 | 1 | 100 | 0 | 0 | 0 | 6.5 | 1.015 | 500 |
| 136 | Study group - male | 10 | 4 | 1 | 100 | 0 | 25 | 0 | 7 | 1.015 | 500 |
| 137 |  | 0 | 4 | 1 | 100 | 0 | 25 | 0 | 9 | 1.005 | 500 |
| 138 |  | 0 | 8 | 2 | 100 | 0 | 25 | 0 | 8 | 1.015 | 500 |
| 139 |  | 0 | 8 | 1 | 100 | 0 | 0 | 0 | 8 | 1.015 | 500 |
| 140 |  | 50 | 8 | 2 | 100 | 0 | 0 | 0 | 8 | 1.015 | 25 |

**Detailed data for pyrogenicity test**

Table S13. Pyrogenicity test results.

| Rabbit No. | Weight [g] | Volume Injected [mL] | Temperature Before Injection [°C] | | | | Initial Temperature [°C] |
| --- | --- | --- | --- | --- | --- | --- | --- |
|  |  |  | 1.5h | 1.0h | 0.5h | 0.0h |  |
| 26 | 3250 | 32.5 | 38.6 | 38.7 | 38.8 | 38.8 | 38.8 |
| 27 | 3970 | 39.7 | 38.9 | 38.8 | 38.7 | 38.8 | 38.75 |
| 28 | 3380 | 33.8 | 38.4 | 38.4 | 38.5 | 38.4 | 38.45 |
| Rabbit No. | Temperature After Injection [°C] | | | | | | Temperature Rise [°C] |
|  | 0.5h | 1.0h | 1.5h | 2.0h | 2.5h | 3.0h |  |
| 26 | 38.9 | 38.8 | 38.8 | 38.6 | 38.7 | 38.7 | 0.1 |
| 27 | 38.6 | 38.7 | 38.7 | 38.7 | 38.5 | 38.5 | -0.05 |
| 28 | 38.3 | 38.2 | 38.2 | 38.3 | 38.3 | 38.2 | -0.15 |
|  |  |  |  | Total: 3 rabbits | | | 0.1 |


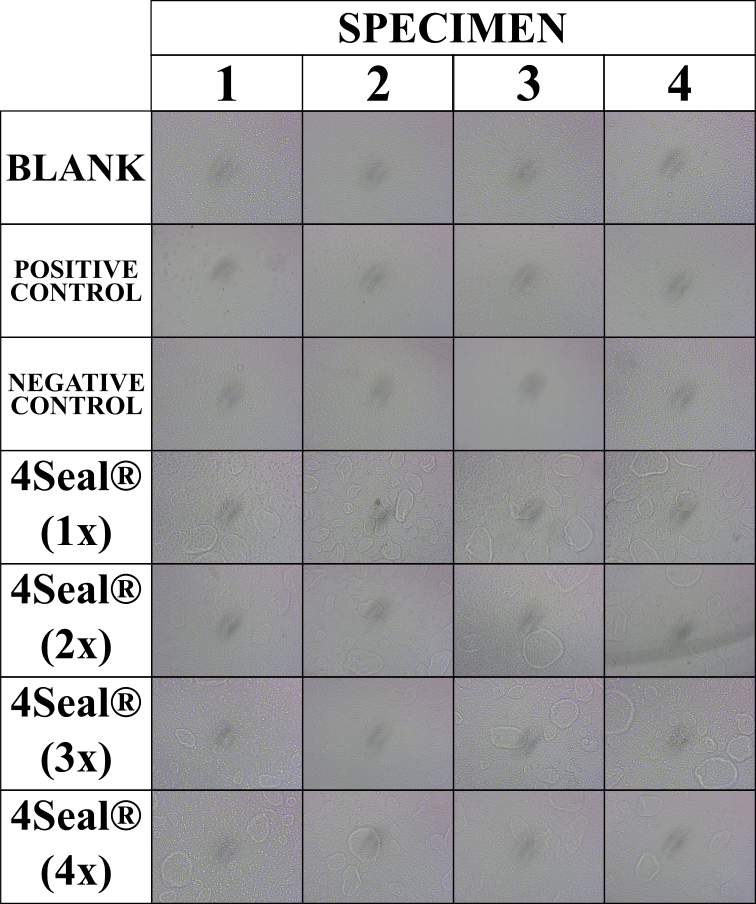


Figure S1. MTT Cytotoxicity of 4Seal® Hemostatic Powder.

Table S14. Result of one-way ANOVA with Bonferroni's multiple comparisons test for MTT Cytotoxicity of 4Seal® Hemostatic Powder

| **Bonferroni's multiple comparisons test** | **Mean Diff.** | **Summary** | **Adjusted P Value** |
| --- | --- | --- | --- |
| 4SEAL® (1x) vs. Positive control | 100.2 | **** | <0.0001 |
| 4SEAL® (2x) vs. Positive control | 102.2 | **** | <0.0001 |
| 4SEAL® (3x) vs. Positive control | 101.2 | **** | <0.0001 |
| 4SEAL® (4x) vs. Positive control | 96.73 | **** | <0.0001 |
| 4SEAL® (1x) vs. Negative control | 8.875 | * | 0.0173 |
| 4SEAL® (2x) vs. Negative control | 10.88 | ** | 0.0029 |
| 4SEAL® (3x) vs. Negative control | 9.875 | ** | 0.0071 |
| 4SEAL® (4x) vs. Negative control | 5.375 | ns | 0.3527 |


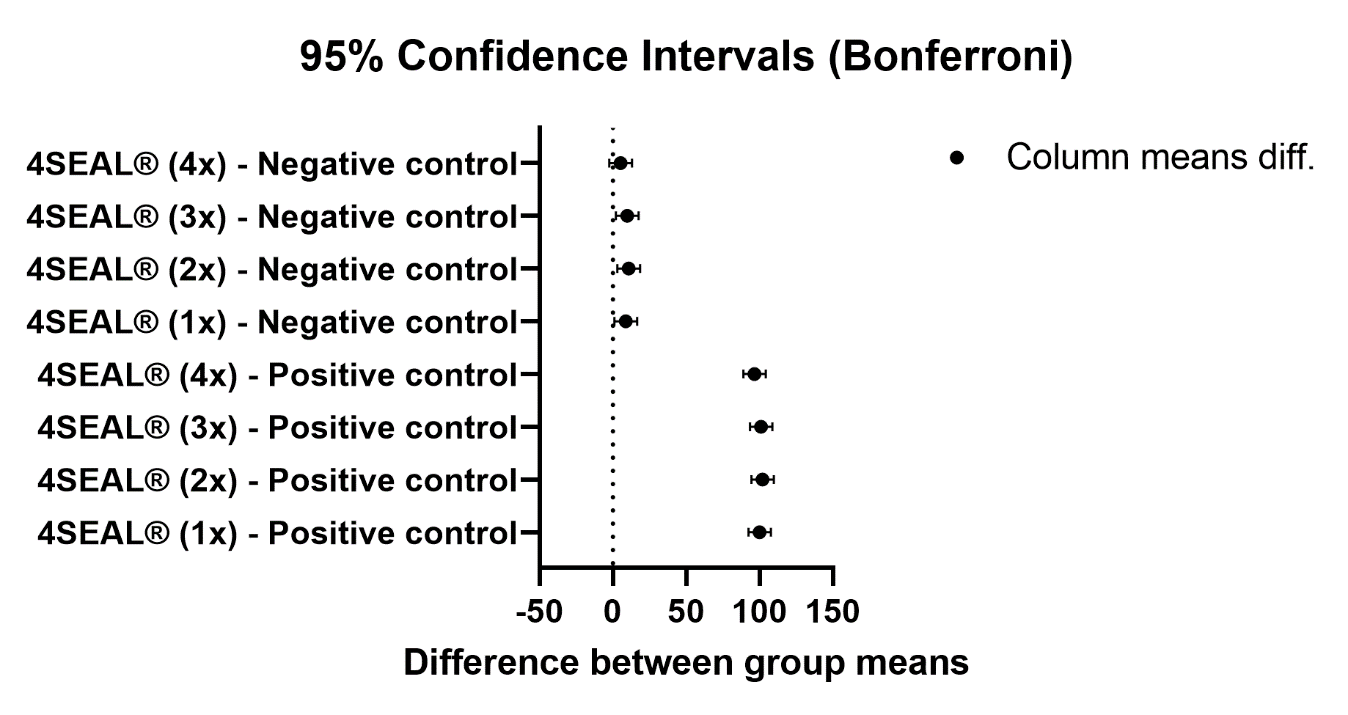


Table S15. Result of one-way ANOVA with Bonferroni's multiple comparisons test for BrdU analysis

| **Bonferroni's multiple comparisons test** | **Mean Diff.** | **Summary** | **Adjusted P Value** |
| --- | --- | --- | --- |
| Negative control vs. Positive control | -0.07200 | **** | <0.0001 |
| Negative control vs. 4SEAL® | -0.01887 | ns | 0.1018 |
| Positive control vs. 4SEAL® | 0.05313 | **** | <0.0001 |


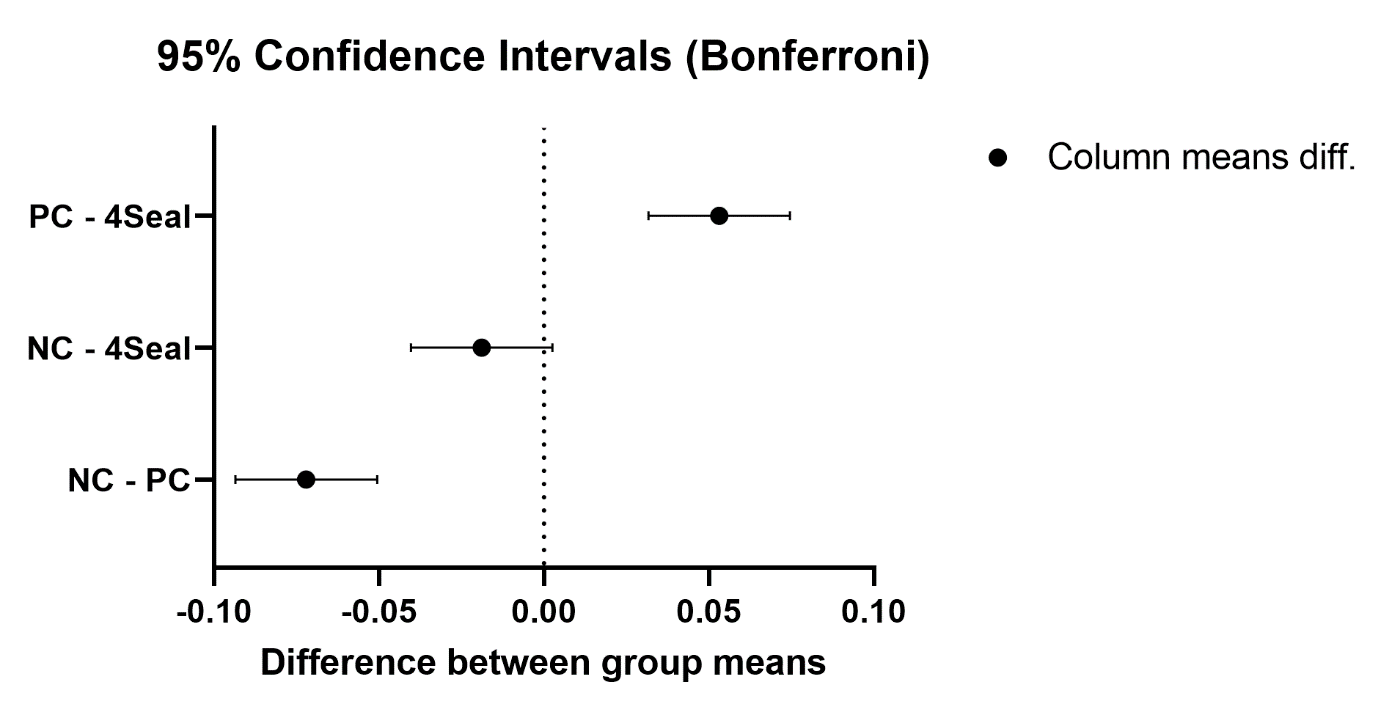

Supplement: Supplementary file 1 — Additional file 1. [file 40824_2022_258_MOESM1_ESM.docx]
